# The E^2.65^A mutation disrupts dynamic binding poses of SB269652 at the dopamine D2 and D3 receptors

**DOI:** 10.1371/journal.pcbi.1005948

**Published:** 2018-01-16

**Authors:** Ravi Kumar Verma, Ara M. Abramyan, Mayako Michino, R. Benjamin Free, David R. Sibley, Jonathan A. Javitch, J. Robert Lane, Lei Shi

**Affiliations:** 1 Computational Chemistry and Molecular Biophysics Unit, Molecular Targets and Medications Discovery Branch, National Institute on Drug Abuse—Intramural Research Program, National Institutes of Health, Baltimore, Maryland, United States; 2 Molecular Neuropharmacology Section, National Institute of Neurologic Disorders and Stroke, National Institutes of Health, Bethesda, Maryland, United States; 3 Departments of Psychiatry and Pharmacology, College of Physicians and Surgeons, Columbia University, New York, New York, United States; 4 Division of Molecular Therapeutics, New York State Psychiatric Institute, New York, New York, United States; 5 Drug Discovery Biology, Department of Pharmacology and Medicinal Chemistry, Monash Institute of Pharmaceutical Sciences, Monash University (Parkville campus), Parkville, Victoria, Australia; UNC Charlotte, UNITED STATES

## Abstract

The dopamine D2 and D3 receptors (D2R and D3R) are important targets for antipsychotics and for the treatment of drug abuse. SB269652, a bitopic ligand that simultaneously binds both the orthosteric binding site (OBS) and a secondary binding pocket (SBP) in both D2R and D3R, was found to be a negative allosteric modulator. Previous studies identified Glu^2.65^ in the SBP to be a key determinant of both the affinity of SB269652 and the magnitude of its cooperativity with orthosteric ligands, as the E^2.65^A mutation decreased both of these parameters. However, the proposed hydrogen bond (H-bond) between Glu^2.65^ and the indole moiety of SB269652 is not a strong interaction, and a structure activity relationship study of SB269652 indicates that this H-bond may not be the only element that determines its allosteric properties. To understand the structural basis of the observed phenotype of E^2.65^A, we carried out molecular dynamics simulations with a cumulative length of ~77 μs of D2R and D3R wild-type and their E^2.65^A mutants bound to SB269652. In combination with Markov state model analysis and by characterizing the equilibria of ligand binding modes in different conditions, we found that in both D2R and D3R, whereas the tetrahydroisoquinoline moiety of SB269652 is stably bound in the OBS, the indole-2-carboxamide moiety is dynamic and only intermittently forms H-bonds with Glu^2.65^. Our results also indicate that the E^2.65^A mutation significantly affects the overall shape and size of the SBP, as well as the conformation of the N terminus. Thus, our findings suggest that the key role of Glu^2.65^ in mediating the allosteric properties of SB269652 extends beyond a direct interaction with SB269652, and provide structural insights for rational design of SB269652 derivatives that may retain its allosteric properties.

## Author summary

G protein-coupled receptors (GPCRs) are targets of more than 25% of prescription drugs on the market. Due to their critical roles in human physiology, competitive modulation of these receptors has been found to be associated with many undesired side effects. Allosteric modulation holds the promise of retaining normal receptor function and improving selectivity. However, the underlying molecular mechanisms of the allosteric modulation of GPCRs have remained largely uncharted. The dopamine D2-like receptors have been implicated in voluntary movement, reward, sleep, learning, and memory. Based on previous experimental findings, we computationally characterized the binding of a negative allosteric modulator of dopamine D2 and D3 receptors, and revealed the dynamic binding mode of this modulator in a secondary binding pocket (SBP) of the receptors. Our results highlight the key role of a Glu in mediating the allosteric properties of the modulator by shaping the dynamically formed SBP, and shed light on rational design and optimization of allosteric modulators of GPCRs.

## Introduction

G protein-coupled receptors (GPCRs) represent one of the largest protein families, and regulate a myriad of physiological processes in response to diverse chemical or environmental stimuli [1]. Among this family, members of the dopamine D2-like receptor subgroup (consisting of dopamine D2 receptor (D2R), D3R, and D4R) have been implicated in various physiological functions, including voluntary movement, reward, sleep, learning, and memory [2]. Previous studies have established dopamine D2-like receptors as important therapeutic targets for a variety of neuropsychiatric disorders as well as for the treatment of drug addictions [2, 3]. Over the last two decades, significant efforts have been made towards understanding the structure-function relationships of these receptors [4–6]. Despite this success, the high sequence identity within the subgroup presents a formidable challenge for selective drug development [7].

In recent years, several bitopic ligands that target both the orthosteric binding site (OBS) and a secondary “allosteric” binding site in GPCRs have been developed to achieve subtype specificity, improve binding affinity, and lead to a reduction in the side effects compared to orthosteric ligands [8]. Whereas most bitopic ligands show competitive behavior against other ligands that bind the OBS [8], SB269652, a bitopic ligand for D2R and D3R, has been shown to act as an allosteric modulator at both receptors [9–12]. SB269652 is composed of a tetrahydroisoquinoline (THIQ) and an indole-2-carboxamide moiety, connected by a cyclohexyl linker in trans orientation. Molecular modeling of SB269652 in D2R showed that the THIQ moiety binds in the OBS and forms an ionic interaction with Asp^3.32^ (superscripts denote Ballesteros-Weinstein numbering [13]), while the indole-2-carboxamide moiety protrudes into a secondary binding pocket (SBP) formed by the extracellular portions of transmembrane segments (TMs) 2 and 7. The pose in the SBP establishes a hydrogen bond (H-bond) between the N atom of the indole-2-carboxamide and Glu^2.65^ [10]. An N-methyl indole-2-carboxamide derivative of SB269652 that is no longer able to make this interaction displayed competitive behavior [14], consistent with an alteration in the binding of the ligand in the SBP. Derivatives based on the indole-2-carboxamide moiety, *N*-isopropyl-1*H*-indole-2-carboxamide and *N*-butyl-1*H*-indole-2-carboxamide, were recently found to display allosteric pharmacology in D2R and D3R, respectively [12, 15], which suggest that the SBP near TMs 2 and 7 is indeed an allosteric binding site. In addition, SB269652 was inferred to mediate negative allosteric modulation through a dimer interface of D2R [10]. Mutagenesis experiments implicated Glu^2.65^ near the proposed TM1 dimer interface of D2R [16] as a key determinant for the activity of SB296652, as replacement of this residue with alanine caused a decrease in both SB269652 affinity and negative cooperativity [10]. Similar disruption by the E^2.65^A mutation of SB269652 binding affinity was also observed at D3R.

However, as the proposed H-bond between Glu^2.65^ and the indole moiety of SB269652 is not a strong interaction and the E^2.65^A mutation did not change the pharmacological profile of SB269652 from allosteric to competitive, the H-bond may not be the only element to determine the allosteric properties [10, 14, 15]. Indeed, our structure activity relationship (SAR) studies also suggested that the size and lipophilicity of the indole-2-carboxamide moiety were also determinants of allosteric pharmacology [14]. Thus, another impact of the E^2.65^A mutation, such as the potentially altered size and shape of the SBP in response to the mutation, may also contribute to the decrease in affinity and negative cooperativity. In the present study, we carried out extensive molecular dynamics (MD) simulations to characterize differences in the binding modes of SB269652 in D2R or D3R, and the impact of the E^2.65^A mutation. Our results elucidate important mechanistic details of the role of Glu^2.65^ in the SBP-mediated change in binding affinity and negative cooperativity.

## Results

### Overview of MD simulations

We carried out comparative MD simulations of four conditions: D2R and D3R wild-type (WT) and their E^2.65^A mutants bound to SB269652. The D2R models in complex with SB269652 were derived from our previous study [10], whereas the starting poses of SB269652 in our D3R models are similar to those in D2R models (see Methods). The first set of simulations was followed by multiple rounds of additional simulations, in which we collected more trajectories for the under-sampled microstates based on the results of the Markov state model (MSM) analysis [17, 18] (see Methods). In total, we collected 145 MD trajectories with a cumulative length of 76.5 μs (Table 1).

**Table 1:** Summary of simulated conditions and simulation lengths.

Receptor	Condition	Number of trajectories	Total length (μs)
D2R	WT	37	18.0
	E^2.65^A	37	21.3
D3R	WT	36	21.3
	E^2.65^A	35	15.9
Total		145	76.5

### The secondary pharmacophore of SB269652 is in a dynamic equilibrium in the SBP of D2R and D3R WT

**Fig 1:**
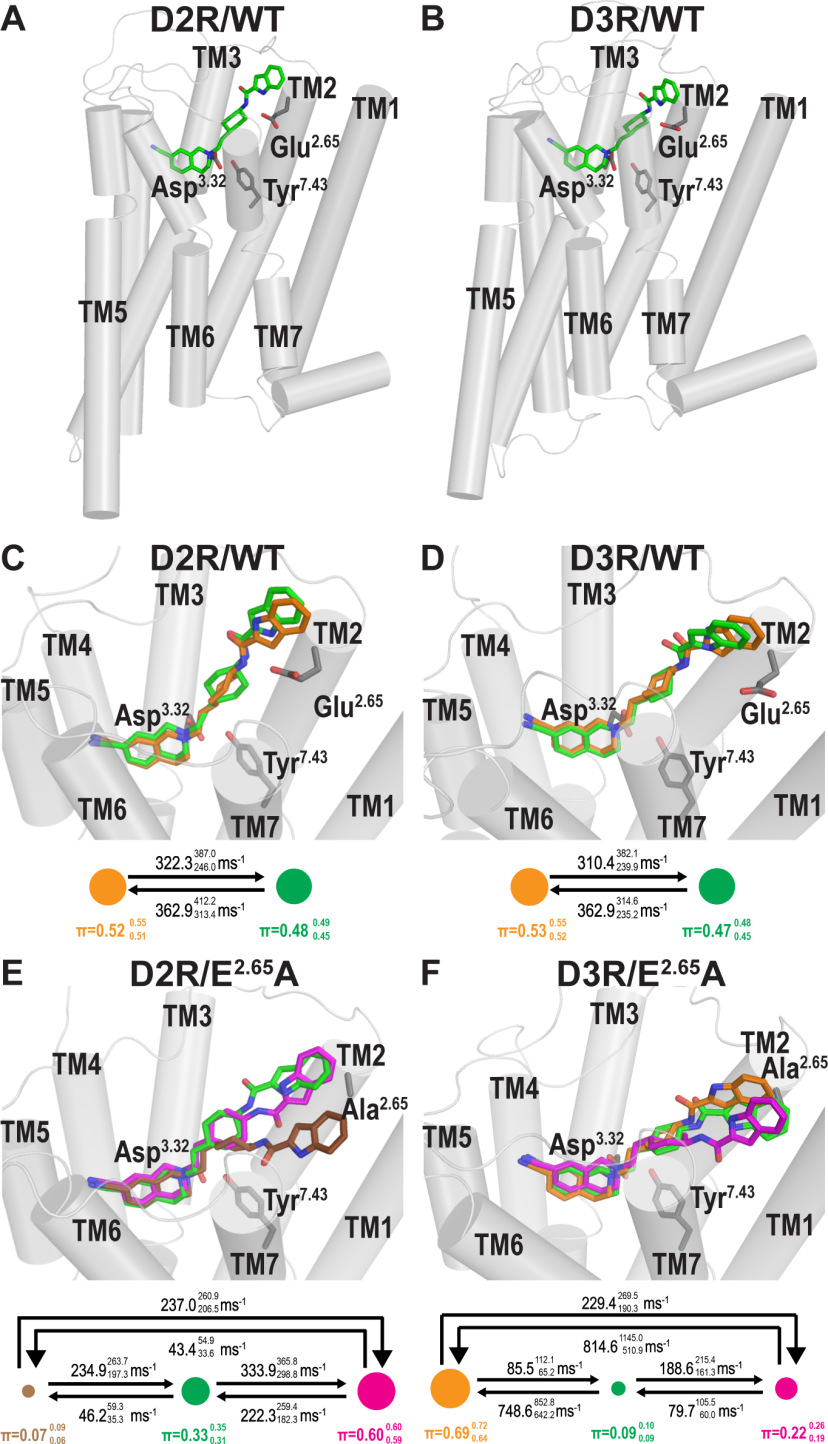
The SP of SB269652 is in dynamic equilibria of binding modes at D2R and D3R. Panels **A** and **B** show the binding modes of SB269652 at D2R/WT and D3R/WT that H-bond with Glu^2.65^. Panels **C** and **D** are zoom-in views of **A** and **B** respectively, with additional binding modes of the SP identified by MD simulations and MSM analysis. Our MSM analysis identified two MSs of SP binding (shown in green and orange). The area of each of the spheres representing a MS is proportional to its equilibrium probability (π); the transition rates between the MSs are shown above the arrows connecting them. Panels **E** and **F** show the binding modes of SB269652 at mutant D2R/E^2.65^A and D3R/E^2.65^A constructs, from the same viewing angles as those in panels **C** and **D**. As demonstrated by the results of MSM analysis, the mutation not only disrupts the equilibria of MSs observed in WT (Panel **C** and **D**), but also results in distinct binding modes of the SP (brown and magenta MSs for D2R/E^2.65^A, and magenta MS for D3R/E^2.65^A). The values from the maximum likelihood Bayesian Markov model and the upper and lower 1σ confidence intervals (in superscript and subscript, respectively) for π and the transition rates from 500 Bayesian Markov model samples are shown. Molecular graphics was generated using PyMOL (version 1.7.6.5, Schrödinger, LLC).

**Fig 2:**
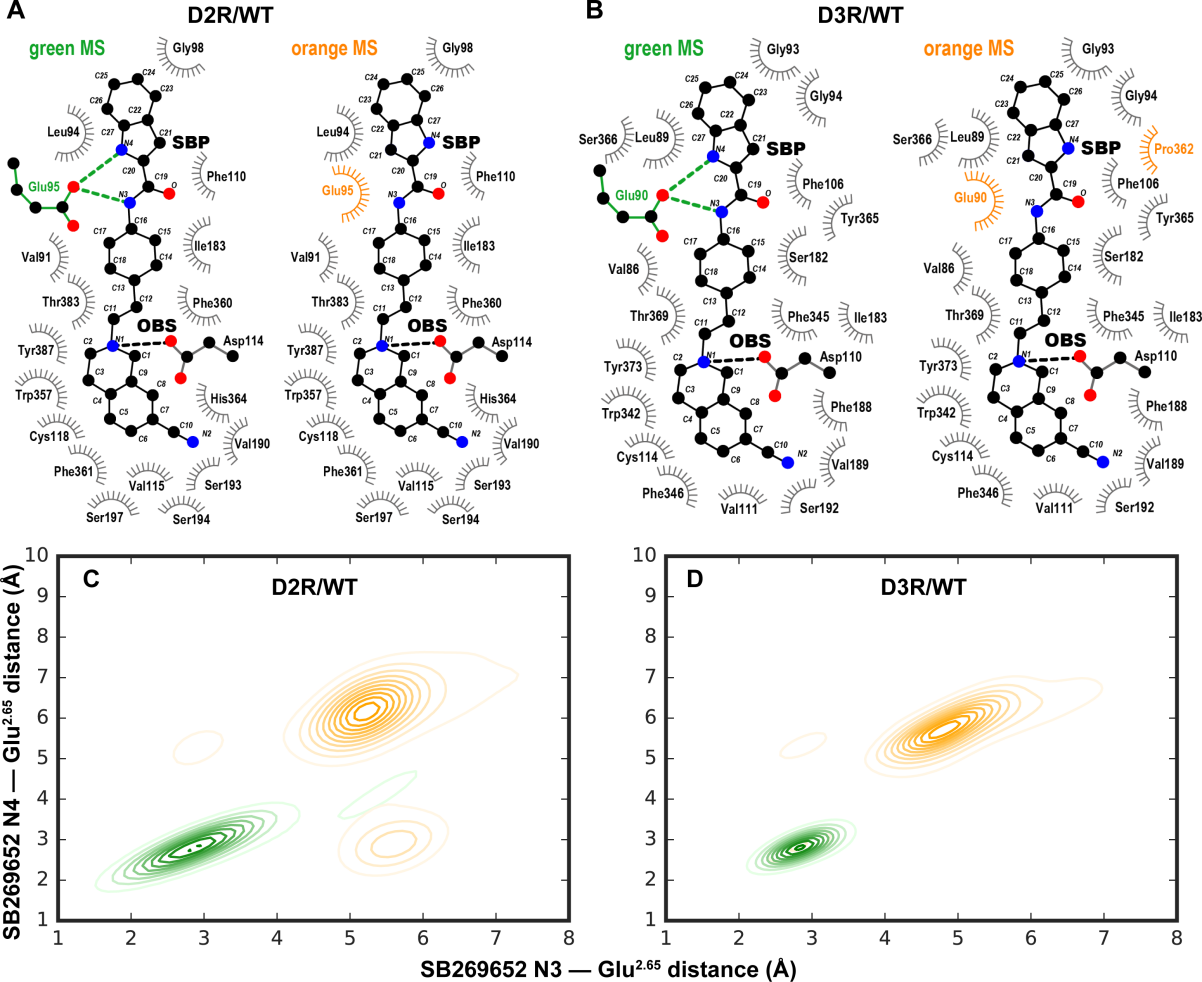
Different binding modes of the SP occupy similar space in each receptor. Panels **A** (D2R/WT) and **B** (D3R/WT) show the protein residues within 5 Å of the heavy atoms of SB269652 (interaction frequency >65%, S1 Table) for the green and orange MSs. Interacting residues shared between the two MSs are shown in grey; residues forming unique interactions in one of the MSs are colored accordingly. The green MSs form direct H-bonds and the orange MSs form non-polar interactions with Glu^2.65^ in both D2R/WT and D3R/WT, but the SP in green and orange MSs occupy similar spaces. Ligand interaction plots were generated using LigPlot+ [19]. The shortest distance between the N3 atom of SB269652 and the carboxyl oxygen atoms of Glu^2.65^ is plotted against that between N4 and those oxygen atoms in each MS of D2R/WT (Panel **C**) and D3R/WT (Panel **D**), showing the similar interaction patterns of the two MSs in the two receptors.

Similar to our previous study [10], in the resulting conformations from our extensive MD simulations, the primary pharmacophore (PP) of SB269652, the THIQ moiety, forms a salt bridge with the carboxyl group of Asp^3.32^ in both D2R and D3R, a key component of ligand binding to aminergic receptors [7]. The secondary pharmacophore (SP), which consists of an indole-2-carboxamide moiety, attached to the PP through a trans-cyclohexylene linker, shows significant dynamics in all our simulated conditions (Fig 1). To characterize the dynamics of the SP poses, we performed MSM analysis to identify the thermodynamic populations of the SB269652 binding poses and to calculate the kinetics of transitions between these populations. Specifically, we used 12 distances between the nitrogen atoms of SB269652 and the Cβ atoms of selected binding-site residues and 4 intra-ligand measures as the input features (see Methods and S1 Fig). The analysis identified two metastable states (MSs) with similar equilibrium probabilities of 52% and 48% in D2R/WT (shown as orange and green spheres in Fig 1C). In the green MS, SB269652 forms two H-bonds to Glu^2.65^ with both the indole N4 and amide N3 atoms as we described previously [10] (Fig 2A and 2C). However, in the orange MS, the H-bond between the N4 atom and Glu^2.65^ is lost as N4 reorients toward the extracellular side (Fig 1C). In addition, whereas N3 is in a similar orientation as in the green MS, it has significantly reduced propensity to form a H-bond with Glu^2.65^ (Fig 2A and 2C).

In contrast to the observed dynamics of the SP among different MSs, in both green and orange MSs, the PP is stable and the salt-bridge interaction between the charged N1 nitrogen in the PP and the key binding-site residue Asp^3.32^ remained intact (S2A Fig), suggesting that the strong salt-bridge interaction deters the dynamics of the SP from propagating to the PP, although we have found that the poses of the PP and SP of bivalent ligands can affect each other [10, 20, 21].

For D3R/WT, we found that the two states identified by the MSM analysis are similar to those in D2R/WT, in terms of the orientations of the indole-2-carboxamide moiety of SB269652, relative to Glu^2.65^. Interestingly, in D3R/WT the orange MS in which the N4 atom of SB269652 faces toward the extracellular side also has a slightly higher equilibrium probability (53%) than the green MS (47%) with the N4 atom interacting with Glu^2.65^ (Fig 1D). Similar to D2R/WT, the PP is stable in D3R/WT as well, with an intact interaction between the N1 nitrogen and Asp^3.32^ (S2B Fig).

### The divergent poses of SB269652 in the OBS of D2R and D3R

**Fig 3:**
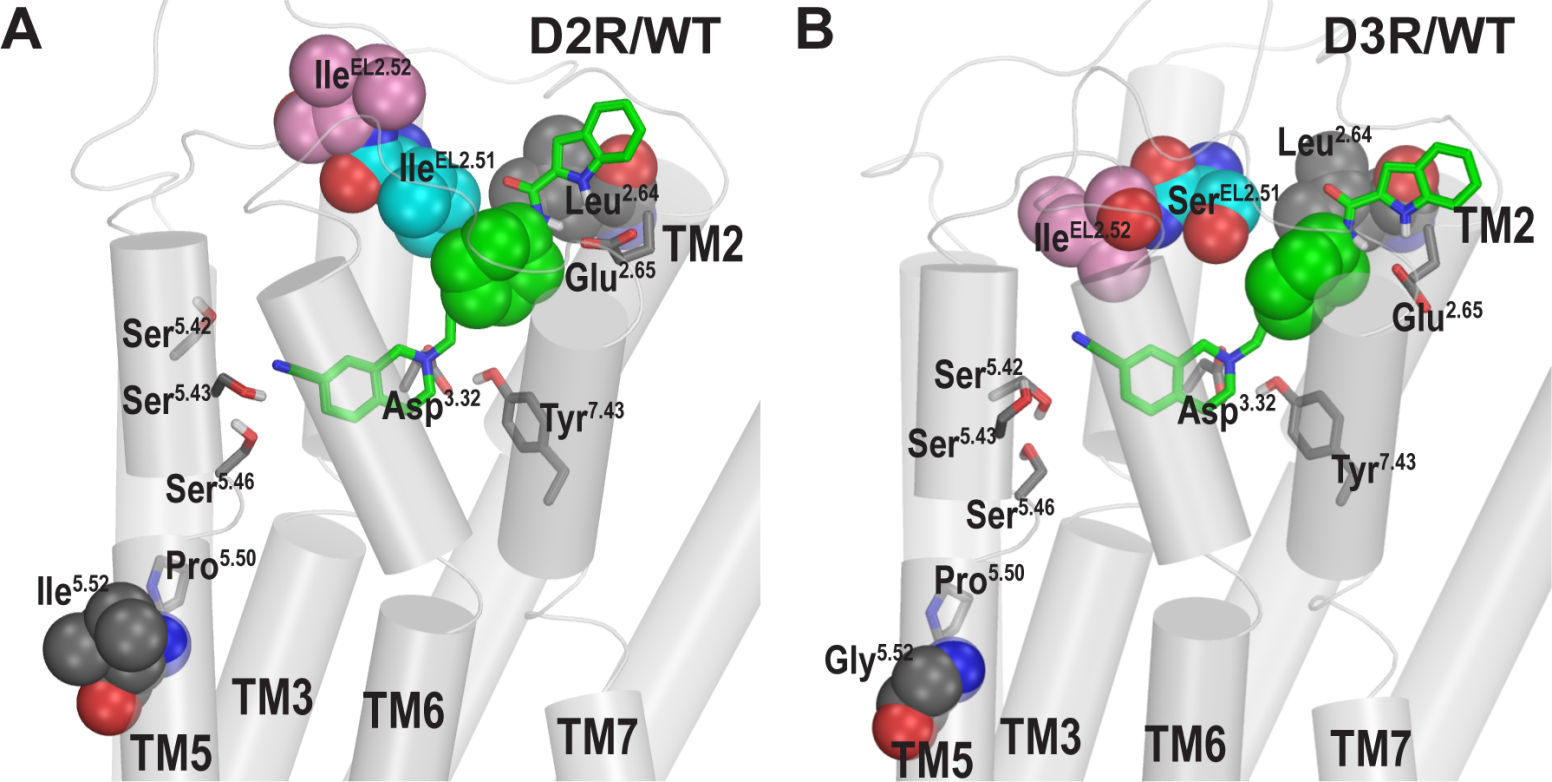
Divergent interactions of SB269652 with EL2 and TM5 in D2R and D3R. Ile^EL2.51^ in D2R forms direct hydrophobic interactions with the cyclohexyl linker of SB269652 and Leu^2.64^ (**A**), while the aligned Ser^EL2.51^ in D3R cannot (**B**), resulting in different orientations of Ile^EL2.52^ in these two receptors. In addition, the divergence in EL2 and near the proline-kink of TM5 (position 5.52) contributes to the different interactions of the serines on top of TM5 with the cyano group of SB269652 (see text). Molecular graphics was generated using PyMOL Molecular Graphics System (version 1.7.6.5, Schrödinger, LLC).

Although the PP is stable in both D2R and D3R, we observed subtly different poses in the OBS of these two receptors. Comparing the representative poses of SB269652 at D2R and D3R, we noticed different interactions between the THIQ moiety and residues from extracellular loop 2 (EL2). Specifically, the subtle divergence of these two receptors at the interface between EL2 and EL1-TM2 accommodates the cyclohexyl linker of SB269652 slightly differently, and this divergence appears to correlate with drastically different orientations of the conserved Ile at the EL2.52 position (second residue after the conserved disulfide-bonded Cys in EL2): while Ile183^EL2.52^ in D3R forms a favored hydrophobic interaction with the THIQ moiety in the OBS, Ile184^EL2.52^ in D2R points upwards and is not in contact with SB269652 (Fig 3 and S1 Table). Such a difference is consistent with the results of our per-residue decompositions of the MM/GBSA binding energy calculations of the representative D2/WT and D3/WT frames, in which Ile^EL2.52^ contributed favorably to binding of SB269652 at D3R but not at D2R.

In addition, we found that SB269652 interacts with Ser193^5.42^, Ser194^5.43^, and Ser197^5.46^ in D2R, while it only interacts with Ser192^5.42^ in D3R. This is likely due to the divergence in both EL2 and TM5 between D2R and D3R –in addition to the divergent EL2.51 and EL2.53 positions in EL2, TM5 is divergent at position 5.52 (Ile203^5.52^ in D2R and Gly202^5.52^ in D3R) near the proline^5.50^-induced kink (Fig 3).

Previously, it was found that SB269652 had more than 10-fold higher binding affinity at D3R than at D2R, and a chimera mutagenesis study that swapped the D2R and D3R segments identified EL2 and TM5 to be important for the different binding affinities [9]. Thus, our findings of the divergent poses of SB269652 in the OBS of D2R and D3R are highly consistent with these results.

### The E^2.65^A mutation alters the dynamics of the SP of SB269652 in the SBP

In comparison to D2R/WT, our MSM analysis identified 3 MSs for the D2R/E^2.65^A condition. One MS of D2R/E^2.65^A is similar to the green MS of D2R/WT; however, given the absence of the H-bond between the indole-2-carboxamide moiety and Ala^2.65^, the indole ring of SB269652 in the green MS of D2R/E^2.65^A tends to be more parallel to the membrane compared to in D2R/WT (Fig 1E). In the dominant new pose of the SP in the D2R/E^2.65^A condition (magenta MS in Fig 1E, which has an equilibrium probability of 60%), both the amide N3 and indole N4 atoms face toward the extracellular side, but the amide O atom faces the intracellular side, which is rarely observed in D2R/WT (Fig 1C and 1E). Interestingly in D3R/E^2.65^A, the three MSs we identified (Fig 1F) have significant similarity to those three in the D2R/E^2.65^A, in terms of the distances of N3 and N4 to Ala^2.65^ (S3 Fig). Even though the orange MS is the most dominant MS (69%) in D3R/E^2.65^A instead of the magenta MS in D2R/E^2.65^A (Fig 1F), in both mutant receptors, N4 of SB269652 has a similar tendency to face away from Ala^2.65^.

### The E^2.65^A mutation affects the size and shape of the SBP in both D2R and D3R

**Fig 4:**
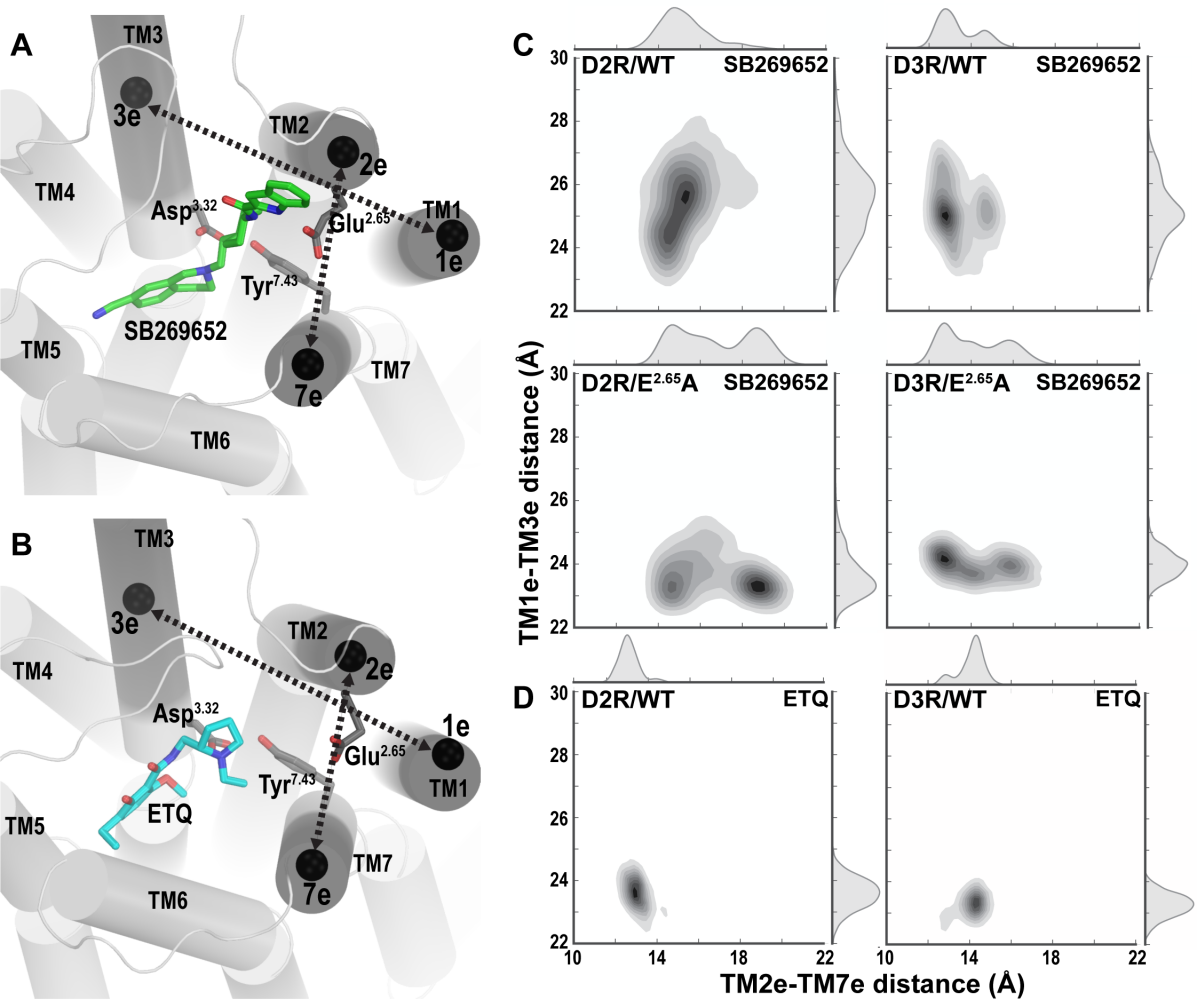
Comparative conformational analysis shows significant changes induced by E^2.65^A in the SBP. The SBP is occupied in the presence of SB269652 (**A**) but not eticlopride (ETQ, **B**), and is enclosed by the TM subsegments TM1e, TM2e, TM3e, and TM7e. The centers of mass for each of these TM subsegments are shown as black spheres. Black dashed arrows indicate the distances measured in (**C**) and (**D**) for two TM subsegment pairs. The molecular graphics was generated using PyMOL (version 1.7.6.5, Schrödinger, LLC). (**C**) Distributions of distances between TM2e-TM7e are plotted against those of TM1e-TM3e distances for all the SB269652-bound conditions, showing larger TM2e-TM7e distances and smaller TM1e-TM3e distances in the E^2.65^A conditions of both receptors. (**D**) The distributions of these distances for the ETQ-bound conditions, when the SBP is not occupied. The MD simulation data of the ETQ-bound conditions were taken from [20].

We hypothesized that in addition to the H-bonds between the indole-2-carboxamide moiety of SB269652 and Glu^2.65^, another key to understanding the significance of the E^2.65^A mutation on the allosteric action of SB269652 lies in conformational changes resulting from this mutation. Our structural analysis identified marked conformational differences between the D2R/WT and D2R/E^2.65^A conditions bound with SB269652, in the SBP consisting of TM1e, TM2e, TM3e, and TM7e subsegments (see S2 Table for the division of subsegments [20, 22]). These differences were characterized by a significantly larger TM2e-TM7e distance and a shorter TM1e-TM3e distance in D2R/E^2.65^A as compared to the D2R/WT condition, demonstrating the altered size and shape of the SBP in the mutant construct (Fig 4). Interestingly, the occupation of the SBP by the SP of SB269652 in D2R/WT increased both TM2e-TM7e and TM1e-TM3e distances (Fig 4C) compared to the D2R/WT condition equilibrated with eticlopride (Fig 4B and 4D), a ligand that predominantly occupies the OBS and does not protrude into the interface between TMs 2 and 7. Thus it appears that the SBP is dynamically formed to accommodate the SP of SB269652, and that the E^2.65^A mutation ablates the ability of SB269652 to increase the distance of TM1e-TM3e through the interaction of its SP with the SBP.

A similar enlargement of the SBP by SB2696952 was observed for D3R/WT as well (Fig 4C and 4D). Comparing the two D3R conditions bound with SB269652, the E^2.65^A mutation results in larger TM2e-TM7e and smaller TM1e-TM3e distances, similar to the observations for the D2R (Fig 4C).

In both D2R and D3R, Glu^2.65^ of TM2e faces Ser^7.36^ of TM7e, and we found that the disruption of this polar interface by the E^2.65^A mutation contributes to the larger TM2e-TM7e distances. However, Glu^2.65^ and Ser^7.36^ have a significant probability to form a H-bond in D3R/WT (green MS, 54.7±0.6%; orange MS, 51.7±1.8%, for the dataset used in Fig 2) but not in D2R/WT (green MS, 14.3±0.5%; orange MS, 9.0±1.3%). Thus, the TM2e-TM7e distance appears to be larger in both the D2R/WT and D2R/E^2.65^A conditions than in D3R/WT and D3R/E^2.65^A (Fig 4C), likely due to the shorter EL1 in D2R, consistent with our previous observations and with differences in the tendencies of this interface to accommodate the SP of the bitopic ligands [23].

**Fig 5:**
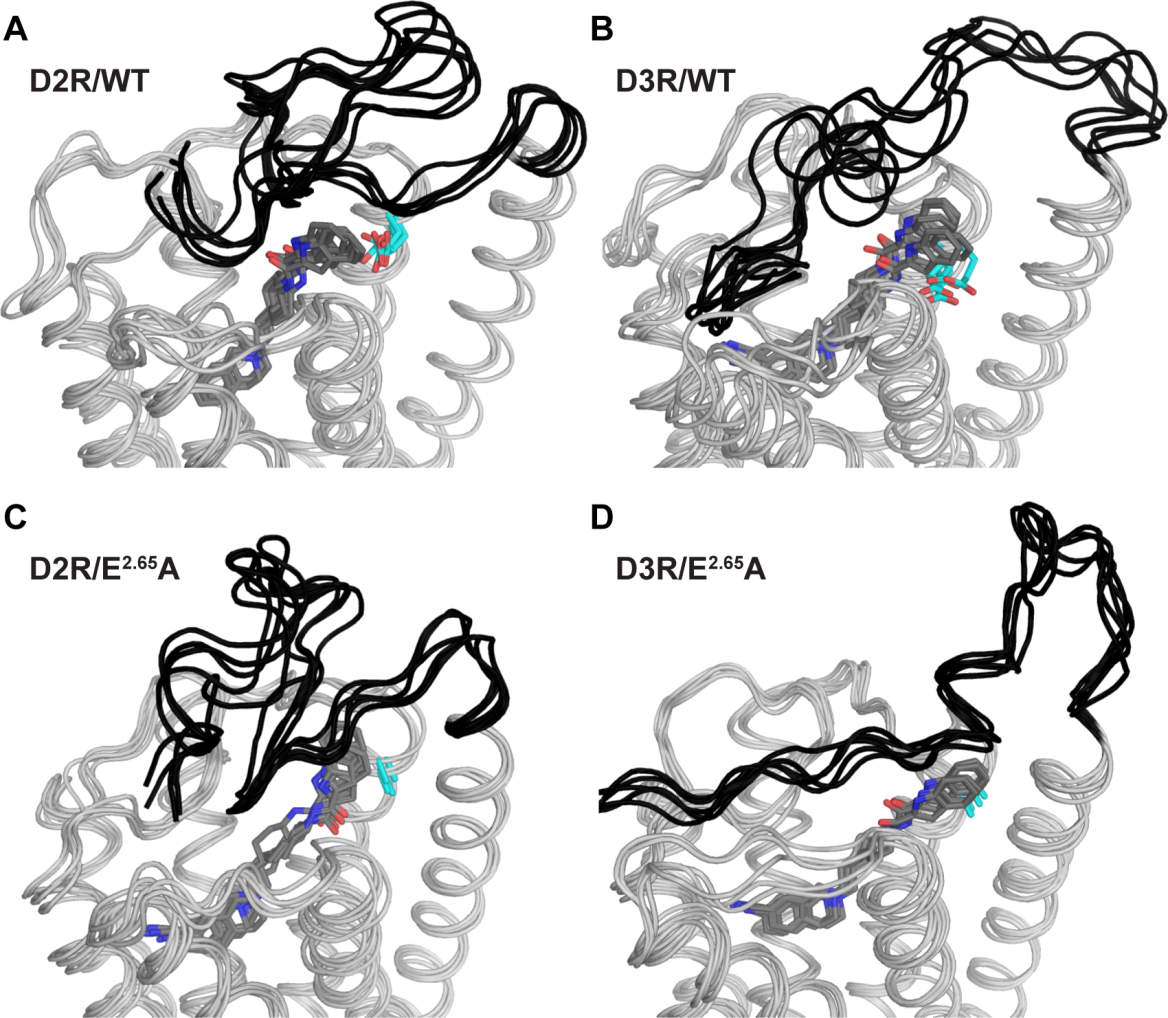
The E^2.65^A mutation affects the conformational dynamics of the NT. For each indicated condition, an ensemble of five representative frames of the largest NT cluster for each condition is shown. The N-terminal region is shown in black, and the bound SB269652 is shown as grey sticks (with nitrogen and oxygen atoms colored in blue and red respectively), whereas the residues at the 2.65 position are shown in cyan sticks. Molecular graphics was generated using PyMOL (version 1.7.6.5, Schrödinger, LLC).

The impact of E^2.65^A on the SBP is associated with altered conformations of N terminus (NT) as well. While the NT always adopts flexible loop conformations in our simulations, our loop clustering analysis (see Methods) indicates clearly distinct equilibria and preferences of the loop conformations in different conditions. For the combined analysis of D2R/WT and D2R/E^2.65^A, we found that the mutation significantly shifts equilibrium of the NT conformation towards one of the two most populated clusters shown in WT, and has ~70% occupancy for the dominant magenta MS of D2R/E^2.65^A (Fig 5 and S3 Table). Thus, the NT appears to be more dynamic in D2R/WT and adopts multiple conformations, whereas the E^2.65^A mutation reduces such dynamics. Similarly, we found the most populated cluster in D3R/E^2.65^A has a significant higher population and is significantly different from that of D3R/WT (Fig 5, S3 Table). Interestingly, it appears that residues 9–13 in D3R have a significant tendency to form a helical conformation, whereas in D2R residues 20 and 21 are dominantly in a bend conformation (S4 Fig). In all conditions, the NT bends down and forms a lid over the extracellular vestibule bringing some of the residues in direct contact with the SP of the ligand (S1 Table).

Taken together, our data show that the E^2.65^A mutation alters the shape and size of the SBP in both D2R and D3R, which in turn affects the NT conformation.

## Discussion

In recent years, there have been significant advances in the development of allosteric modulators for GPCRs that have high selectivity and novel modes of action. These modulators may lead to therapeutic agents that have fewer side effects [24–26]. One such an example is SB269652, which acts as a negative allosteric modulator in both D2R and D3R. Whereas our previous studies identified Glu^2.65^ as a critical residue for allosteric modulation of SB269652 [10], our follow-up SAR study suggests that other elements are also involved in determining the allosteric properties of SB269652 [14]. By carrying out comparative MD simulations in combination with MSM analysis of D2R and D3R WT and their E^2.65^A mutants, we sought to comprehensively characterize the binding poses and dynamics of SB269652 and the impact of E^2.65^A mutation on the size and shape of the SBP.

The results of our MD simulations and MSM analysis revealed that in both D2R/WT and D3R/WT, SB269652 has significant probabilities of not forming the H-bonds with Glu^2.65^, and its SP is in dynamic equilibria between two poses, although they essentially occupy the same space in the SBP in each receptor (Figs 1 and 2). These results suggest that the direct H-bond interactions between the ligand SP and Glu^2.65^ are not the only factor that governs the allosteric property of SB269652 in either D2R or D3R. Indeed, the mutation E95^2.65^A did not cause a switch from allosteric to competitive pharmacology, but rather caused a decrease in the affinity and negative cooperativity of SB269652 [10]. The dynamic equilibria of the binding poses for the allosteric moiety of SB269652 is likely a common feature shared by the binding of other bitopic ligands in allosteric pockets–in many cases, such pockets of GPCRs are located at peripheral regions, more exposed to the water milieu, and may have more dynamic and flexible properties than the OBS. While unlikely to be revealed by crystallography, such dynamic features can be readily identified and characterized by extensive MD simulations in combination with MSM analysis.

Our results also indicate that the E^2.65^A mutation significantly alters the dynamic equilibria of the SP of SB269652 in the SBP and results in new poses of the ligand. The new poses (magenta MSs), although not observed in WT for both receptors, are similar to the orange MSs of WT that have the N4 atom facing away from Glu^2.65^ (Figs 1C and 2A). From the perspective of receptor conformation, we found the substitution of the charged and larger Glu^2.65^ residue to a smaller Ala residue significantly affects the packing in the SBP, leading to larger TM2e-TM7e and smaller TM1e-TM3e distances in both receptors (Fig 4). Thus, we propose that the combined impact from both the removal of the H-bonds and the altered SBP is responsible for the decrease in affinity and cooperativity of SB269652 observed in the E^2.65^A mutants. Given the significant role of the size and shape of the SBP in mediating the allosteric properties of SB269652, we can envision that some SB269652 derivatives may have allosteric properties even without the capability of forming H-bonds with Glu^2.65^, as long as they can induce the necessary conformational changes of the SBP. Such conformational changes may be impaired by the E^2.65^A mutation irrespective of whether a ligand has the capacity to H-bond with Glu^2.65^. Of note, our SAR studies reveal that an N-methyl indole-2-carboxamide derivative of SB269652 (MIPS1500) displayed apparently competitive behavior at D2R/WT, but acted as a negative allosteric modulator of dopamine at D2R/E95^2.65^A [10]. While such observations may reflect the inability of this ligand to form a H-bond with Glu^2.65^ as we originally proposed, the addition of a methyl group also adds bulk to the SP. This may change its orientation within the SBP. Thus, by changing the configuration of the SBP, the E95^2.65^A mutation may change the orientation of the N-methyl indole-2-carboxamide moiety of MIPS1500 within the SBP causing the ‘gain’ of allosteric pharmacology. Indeed, the size of the SP has also been shown to be an important determinant of the allosteric pharmacology of SB269652, as derivatives in which the indole moiety was replaced by a pyrrole or proline moiety display apparently competitive pharmacology at the D2R [14]. Such derivatives retain the ability to form an interaction with Glu^2.65^ but lack the lipophilicity and bulk of the indole moiety. Interestingly, our recent mutagenesis studies reveal that, in a similar manner to the N-methyl indole moiety, the pyrrole derivative displays allosteric pharmacology at D2R/E95^2.65^A (Draper Joyce et al., manuscript in preparation). Such observations are consistent with the conformation of the SBP, and the influence of the SP on this conformation, being central to the allosteric pharmacology of SB269652.

Our results also show that the altered size and shape of the SBP in E^2.65^A mutants could bias the NT towards specific conformations, and in the case of D3R, a distinct one from the most populated conformation in the WT. In all conditions, the NT forms a lid over the extracellular vestibule and is in direct interaction with SB269652, suggesting a previously unappreciated role of the NT in ligand binding at D2R and D3R. Indeed, the functional roles of the NT have been documented recently in a few closely related homologs, including the α_1D_-adrenergic [27], 5-HT_2B_ [28] and μ-opioid receptors [29, 30]. By systematically examining all the available high-resolution crystal structures of class-A GPCRs bound to small compounds, we found 9 structures of 6 receptors showing direct interactions (within 5 Å of the heavy atoms) between NT residues and the small-molecule ligands that at least partially occupy the OBS. Interestingly, many of these small-molecule ligands protrude into the interface between TMs 2 and 7 (S5 Fig).

Taken together, our findings highlight the key role of the size and shape SBP, which is determined by Glu^2.65^, in mediating the allosteric properties of SB269652, and provide structural insights for the rational design of SB269652 derivatives that may retain these allosteric properties.

## Methods

### MD simulations

The binding mode of SB269652 at D2R was investigated based on our previous study [10]. Briefly, to acquire a reference binding mode of the PP (tetrahydroisoquinoline (THIQ)) of SB269652 in the high-resolution crystal structure of D3R (PDB code 3PBL [31]), THIQ in the protonated form was first docked into the D3R structure with the induced-fit docking (IFD) protocol [32] implemented in Schrödinger suite (release 2016–1, Schrödinger, LLC: New York, NY). The lowest MM/GBSA energy pose from the largest binding mode cluster was selected as a reference pose for the PP of SB269652 at D3R. Assuming that binding modes of THIQ in the near-identical OBSs of D3R and D2R should be similar, we docked the THIQ into the D2R model [20, 21, 23, 31, 33] and selected a pose that is closest to the THIQ reference pose in the D3R structure. The full-length SB269652 was then docked into the D2R and D3R models by restraining the PP core [21] to the respective THIQ reference poses (with RMSD tolerance for the heavy-atom restraints of < 2.0 Å). To investigate the effect of the E^2.65^A mutation, Glu^2.65^ was mutated to Ala in representative frames from equilibrated WT trajectories, and the charge of the system was neutralized by removing a Na^+^ ion from the water milieu.

MD simulations of the receptor–ligand complexes were performed in the explicit water and 1-palmitoyl-2-oleoylphosphatidylcholine (POPC) lipid bilayer environment using Desmond MD System (version 4.5; D. E. Shaw Research, New York, NY) with the CHARMM36 force field [34–37] and TIP3P water model. The ligand parameters were obtained through the GAAMP server [38], with the initial force field based on CGenFF assigned by ParamChem [39]. The system charges were neutralized, and 150 mM NaCl was added. The average size of the simulation systems was ~110000 atoms.

The protein-membrane relaxation was carried out with a protocol modified from that developed by Schrödinger, LLC. Briefly, the initial energy minimization was followed by equilibration with restraints on all protein and ligand heavy atoms in the beginning for 1 ns, then with restraints only on the protein backbone and ligand heavy atoms for 6 ns. For both the equilibrations and the following unrestrained production runs, we used Langevin constant pressure and temperature dynamical system [40] to maintain the pressure at 1 atm and the temperature at 310K, on an anisotropic flexible periodic cell with a constant-ratio constraint applied on the lipid bilayer in the X-Y plane.

For each condition, we collected several rounds of multiple trajectories following the procedure described below.

### MSM analysis

The MSM analysis was performed using the PyEMMA program (version 2.3.2) [41]. For the input featurizer, we chose the features based on the following considerations to describe the interactions and orientations of SB269652 within the receptor binding sites. The polar and charged interactions between the ligand and protein contribute significantly to ligand binding, while these interactions can be more conveniently defined and characterized by simple geometric measures, compared to hydrophobic and aromatic interactions. For SB269652, the nitrogen atoms are distributed in both the THIQ and indole-2-caboxyamide moieties, so that the dynamics of the entire ligand can be properly characterized using distances between these atoms and protein residues. Therefore, we identified protein residues with their Cβ atoms within 7.0 Å of any of the four nitrogen atoms of the SB269652, and used these Cβ-N distances as input features. To better account for the orientation of the indole-2-caboxyamide moiety of SB269652, two additional intramolecular distances from the N4 atom of indole ring were included in the features—one to the N3 nitrogen and one to the oxygen of the amide bond. Further, we calculated the vectors from the centers of mass of 5- or 6-member rings of the SP to N4, and the projections of these vectors on the axis perpendicular to membrane were included to identify the orientation of the indole ring relative to the plane of the membrane. In total, 16 input features were used (S1 Fig).

The slow linear subspace of the input coordinates was estimated by the time-lagged independent component analysis (TICA) [42, 43] on the combined data set of D2R/WT, D2R/E^2.65^A, D3R/WT, and D3R/E^2.65^A conditions, and a dimension reduction was achieved by projecting on the 4 slowest TICA components (which represent 61.7% of cumulative kinetic variance). k-means clustering was then employed to discretize the simulated subspace and 100 microstates (MIs) were obtained. For a range of numbers of MIs (50, 75, 100, 150, 200, 300), we estimated an MSM for each situation and concluded that the 100-MI MSM performs best based on two analysis. We first calculated scores in terms of variational principle [44, 45], using cross-validation [46] as previously described [47]. This analysis showed the variational scores were comparable for small numbers of MI and decreased when the number of MI is larger than 150. In addition, the 100-MI MSM showed better convergence of the implied time scales (ITS) in terms of the lag times (S6–S8 Figs).

The discretized combined data set was then divided into individual simulated conditions to estimate Bayesian MSMs [48]. The Bayesian sampling was used to compute statistical uncertainties of 500 transition matrix samples at each lag time. Convergence of ITS for all MSMs was achieved at 96 ns lag time (S7 Fig), which was used to estimate Bayesian MSM for each condition. The PCCA++ method [49] implemented in PyEMMA was then used to stitch the MIs into metastable states (MSs). In the resulting MSMs, 2 MSs were assigned to D2R/WT and D3R/WT conditions each, whereas 3 MSs were assigned to D2R/E^2.65^A and D3R/E^2.65^A conditions each. The identity of common MSs between different conditions was determined based on the number of shared MIs between them. Further structural and kinetic analysis was performed using frames from those MIs that have > 70% probability belonging to their respective MS.

In Fig 1, the transition rate between two MSs is the inverse mean first passage time, which is the expected hitting time of one MS when starting from the other MS. The π value denotes the equilibrium probability of a given MS, which is the probability to be in the MS that remains unchanged in the Markov model as time progresses. The transition rate and equilibrium probability were computed as described previously [50].

The validity of the MSMs was assessed using Chapman-Kolmogorov tests (S9 and S10 Figs) which showed that the MSMs estimated at 96 ns were consistent with the simulation data within the 95% confidence interval computed by 500 bootstrapped samples of trajectories. Generally, the Chapman–Kolmogorov test checks if the MSM models estimated at lag time τ can be used to make predictions for the data at longer times *k*τ within statistical error, *i*.*e*., if Eq (1) can be satisfied:

$$P(k\tau )=P^{k}(\tau )$$ (1)

where *P*(τ) is the transition matrix estimated from the data at lag time τ (the Markov model), and *P*(*k*τ) is the transition matrix estimated from the same data at longer lag times *k*τ. In practice, we use *P*(*k*τ) and *P*^*k*^(τ), respectively, to propagate probability starting from one of the metastable states, and measure how much probability ends up in each metastable state [47].

### MSM-guided iterative MD sampling

To adequately and efficiently i) explore the conformational space and ii) sample the transitions between MSs, we developed an iterative MD sampling protocol to guide the simulations to i) the less-than-well-sampled regions and ii) the saddle points on the energy surface that are likely in between MSs. Thus, by taking advantage of the MSM analysis after each round of simulations, we correspondingly identified both i) the single-frame microstates (MIs), and ii) the under-sampled MIs (< 10 frames) that are in between MSs (i.e., the MIs having similar equilibrium probabilities to be in two or more MSs), as the starting points for the next round of simulations. For the selected MIs with more than one frames, we select the representative frames from the more advanced stages of MD simulations. The procedure to identify the MIs satisfying the criteria and to select the frame has been automated with an in-house python script. For the MSM-guided simulations, we collected 300 ns for each trajectory. The simulations were considered to have reached convergence until the biggest change in the equilibrium probabilities for the updated MSMs of each condition was < 5% after including data from the new round of the simulations.

### Conformational analysis

For the identifications of ligand contact residues shown in Fig 2, the results are based on 500 Bayesian Markov model samples 3 frames each from those MIs having > 70% probability of belonging to each MS. For each of the MS we identified residues within 5.0 Å of ligand (heavy atom-heavy atom distances) in D2R/WT and D3R/WT conditions, and the means and standard deviations of three sets of such samplings are shown in S1 Table.

For the sub-segment distance calculations shown in Fig 4, the TMs in both D2R and D3R were divided into subsegments (extracellular, middle, and intracellular) as described in [20] (see S2 Table), the results are based on 3000 Bayesian Markov model samples for each condition with the number of samples for each MS proportional to their equilibrium probability, from those MIs having > 70% probability of belonging to each MS.

### Clustering of the conformations of the N terminus

We performed the clustering analysis of the conformations of the N terminus (NT) using the same dataset extracted for Fig 2 (see above) for each MS in each condition, and combined data sets for one receptor together. The clustering is based on pairwise RMSD of selected NT residues by iteratively excluding the residues with high (> 5.0 Å) root mean squared fluctuation (RMSF). The final clustering results are based on residues 6–20 and 22–30 for D2R and 2–25 for D3R to perform superimpositions and RMSD calculations. The computed RMSD matrix was then subjected to hierarchical clustering using Ward algorithm implemented in SciPy. The number of clusters for each receptor was determined so that the intra-cluster mean pairwise RMSD for each cluster is within 5.0 Å, unless the given cluster has less than 5% of the total frames. The population of each cluster was re-weighted by equilibrium probabilities of the MSs that their members belong to.

The means and standard deviations for the three largest clusters are shown in S3 Table.

## Supporting information

S1 Fig Chemical structure of SB269652 and the input features for MSM analysis. The tetrahydroisoquinoline (PP) and indole-2-carboxamide moiety (SP) of SB269652 are shown in green and pink respectively. COM5 and COM6 refer to the centers of mass of the 5- and 6-member rings of the SP, respectively. The input features for MSM analysis include 12 distances from the nitrogen atoms of SB269652 to the indicated receptor Cβ atoms, 2 intramolecular distances of SB269652, and the projections of the vectors from COM5 or COM6 to N4 along the axis perpendicular to membrane (see Methods).
(PDF)

S2 Fig The salt bridge between the N1 atom of SB269652 and Asp^3.32^ remains stable in all conditions. Distributions of the minimum distances between the N1 nitrogen atom of SB269652 and carboxyl oxygen atoms of Asp^3.32^ for each MS of the indicated conditions are shown. The MSs for each condition are colored according to Fig 1.
(PDF)

S3 Fig Distribution of the distances from the N3 and N4 atoms of SB269652 to the Cβ atom of Ala^2.65^ in mutant receptors. The MSs for each condition are colored according to Fig 1.
(PDF)

S4 Fig Distribution of secondary structure content for each of the N terminal residues. The secondary structures were classified by DSSP v2.0.4 [1]. The boundary between NT and TM1 is indicated by the dotted line.
(PDF)

S5 Fig The high-resolution crystal structures of class-A GPCRs with the NT in contact with small-molecule ligands. The NT is within 5 Å (heavy atom-heavy atom distance) of the small-molecule ligands that at least partially occupy the OBS in the crystal structures of (**A**) Chemokine receptors CCR2 (PDB: 5T1A) [1] and (**B**) CXCR4 (PDB: 3ODU) [2], (**C**) lysophospholipid receptor-1 (PDB: 4Z34) [3], (**D**) μ-opioid receptor (PDB: 5C1M) [4], (**E**) sphingosine 1-phosphate receptor subtype 1(PDB: 3V2Y) [5], and (**F**) cannabinoid receptor CB1 (PDB: 5TGZ, 5U09, 5XR8, and 5XRA) [6–8].
(PDF)

S6 Fig Implied time scales for 50- and 75-microstate MSMs. Implied timescales (ITS) are plotted against lag time. The ITS of the maximum likelihood Bayesian Markov model are shown in solid lines, whereas the means and the 95% confidence intervals (computed by Bayesian sampling) are shown in dashed and shaded areas, respectively. In blue, red, and green, … are the 1^st^, 2^nd^, and 3^rd^, … slowest ITS.
(PDF)

S7 Fig ITS for 100- and 150-microstate MSMs. The color coding is the same as in S6 Fig.
(PDF)

S8 Fig ITS for 200- and 300-microstate MSMs. The color coding is the same as in S6 Fig.
(PDF)

S9 Fig Chapman-Kolmogorov test for D2R/WT and D2R/E^2.65^A. For each of the MSs in our models we initialize the population in that state and compare the evolution of the population predicted from the MSM at given lag times (dashed blue lines) to that measured on the trajectories (black lines) at the same lag times. The shaded areas (light blue) represent 95% confidence intervals computed by bootstrapping. The colors of the MSs are the same as in Fig 1.
(PDF)

S10 Fig Chapman-Kolmogorov test for D3R/WT and D3R/E^2.65^A. See S9 Fig for details.
(PDF)

S1 Table SB269652 interacting residues. The means and standard deviations of the interaction frequencies for each residue were calculated using 3 sets of 500 MSM Bayesian samples, each set containing 1500 frames per MS. Residues having contact frequencies above 25% in at least one of the MS are listed and the contact frequencies above 25% are in bold. OBS and SBP residues are highlighted in cyan and yellow, respectively.
(PDF)

S2 Table Definition of helical subsegments. Ballesteros-Weinstein numbering for the subsegments is given in parenthesis.
(PDF)

S3 Table RMSD based clustering of the N terminus region. 3 sets of 500 Bayesian Markov model samples were subjected to RMSD based clustering of the NT conformation. The percentage populations of the three largest clusters are shown, the populations of these clusters in the most dominant MS in each condition are shown in bold. The numbers in parenthesis refer to cluster ids.
(PDF)

## References

[1] Lagerstrom MC, Schioth HB. Structural diversity of G protein-coupled receptors and significance for drug discovery. Nat Rev Drug Discov. 2008;7(4):339–57. doi: 10.1038/nrd2518 . 18382464 10.1038/nrd2518

[2] Beaulieu JM, Gainetdinov RR. The physiology, signaling, and pharmacology of dopamine receptors. Pharmacol Rev. 2011;63(1):182–217. doi: 10.1124/pr.110.002642 . 21303898 10.1124/pr.110.002642

[3] Heidbreder CA, Newman AH. Current perspectives on selective dopamine D-3 receptor antagonists as pharmacotherapeutics for addictions and related disorders. Addiction Reviews 2. 2010;1187:4–34. doi: 10.1111/j.1749-6632.2009.05149.x. WOS:000277795700001. 20201845 10.1111/j.1749-6632.2009.05149.x PMC3148950

[4] Missale C, Nash SR, Robinson SW, Jaber M, Caron MG. Dopamine receptors: from structure to function. Physiol Rev. 1998;78(1):189–225. doi: 10.1152/physrev.1998.78.1.189 . 9457173 10.1152/physrev.1998.78.1.189

[5] Katritch V, Cherezov V, Stevens RC. Structure-function of the G protein-coupled receptor superfamily. Annu Rev Pharmacol Toxicol. 2013;53:531–56. doi: 10.1146/annurev-pharmtox-032112-135923 ; PubMed Central PMCID: PMCPMC3540149. 23140243 10.1146/annurev-pharmtox-032112-135923 PMC3540149

[6] Beaulieu JM, Espinoza S, Gainetdinov RR. Dopamine receptors—IUPHAR Review 13. Br J Pharmacol. 2015;172(1):1–23. doi: 10.1111/bph.12906 ; PubMed Central PMCID: PMCPMC4280963. 25671228 10.1111/bph.12906 PMC4280963

[7] Michino M, Beuming T, Donthamsetti P, Newman AH, Javitch JA, Shi L. What Can Crystal Structures of Aminergic Receptors Tell Us about Designing Subtype-Selective Ligands? Pharmacological Reviews. 2015;67(1):198–213. doi: 10.1124/pr.114.009944. WOS:000346823400003. 25527701 10.1124/pr.114.009944 PMC4279073

[8] Lane JR, Sexton PM, Christopoulos A. Bridging the gap: bitopic ligands of G-protein-coupled receptors. Trends Pharmacol Sci. 2013;34(1):59–66. doi: 10.1016/j.tips.2012.10.003 . 23177916 10.1016/j.tips.2012.10.003

[9] Silvano E, Millan MJ, la Cour CM, Han Y, Duan LH, Griffin SA, et al The Tetrahydroisoquinoline Derivative SB269,652 Is an Allosteric Antagonist at Dopamine D-3 and D-2 Receptors. Molecular Pharmacology. 2010;78(5):925–34. doi: 10.1124/mol.110.065755. WOS:000283148700016. 20702763 10.1124/mol.110.065755 PMC2981362

[10] Lane JR, Donthamsetti P, Shonberg J, Draper-Joyce CJ, Dentry S, Michino M, et al A new mechanism of allostery in a G protein-coupled receptor dimer. Nature Chemical Biology. 2014;10(9):745–52. doi: 10.1038/nchembio.1593. WOS:000341126800011. 25108820 10.1038/nchembio.1593 PMC4138267

[11] Kumar V, Moritz AE, Keck TM, Bonifazi A, Ellenberger MP, Sibley CD, et al Synthesis and Pharmacological Characterization of Novel trans-Cyclopropylmethyl-Linked Bivalent Ligands That Exhibit Selectivity and Allosteric Pharmacology at the Dopamine D3 Receptor (D3R). J Med Chem. 2017;60(4):1478–94. doi: 10.1021/acs.jmedchem.6b01688 ; PubMed Central PMCID: PMCPMC5325712. 28186762 10.1021/acs.jmedchem.6b01688 PMC5325712

[12] Furman CA, Roof RA, Moritz AE, Miller BN, Doyle TB, Free RB, et al Investigation of the binding and functional properties of extended length D3 dopamine receptor-selective antagonists. European Neuropsychopharmacology. 2015;25(9):1448–61. doi: 10.1016/j.euroneuro.2014.11.013. WOS:000361862500007. 25583363 10.1016/j.euroneuro.2014.11.013 PMC4449328

[13] Ballesteros JA, Weinstein H. [19] Integrated methods for the construction of three-dimensional models and computational probing of structure-function relations in G protein-coupled receptors. In: Stuart CS, editor. Methods in Neurosciences. Volume 25: Academic Press; 1995 p. 366–428.

[14] Shonberg J, Draper-Joyce C, Mistry SN, Christopoulos A, Scammells PJ, Lane JR, et al Structure-activity study of N-((trans)-4-(2-(7-cyano-3,4-dihydroisoquinolin-2(1H)-yl)ethyl)cyclohexyl)-1H-ind ole-2-carboxamide (SB269652), a bitopic ligand that acts as a negative allosteric modulator of the dopamine D2 receptor. J Med Chem. 2015;58(13):5287–307. doi: 10.1021/acs.jmedchem.5b00581 . 26052807 10.1021/acs.jmedchem.5b00581

[15] Mistry SN, Shonberg J, Draper-Joyce CJ, Klein Herenbrink C, Michino M, Shi L, et al Discovery of a Novel Class of Negative Allosteric Modulator of the Dopamine D2 Receptor Through Fragmentation of a Bitopic Ligand. J Med Chem. 2015;58(17):6819–43. doi: 10.1021/acs.jmedchem.5b00585 . 26258690 10.1021/acs.jmedchem.5b00585 PMC10823399

[16] Guo W, Urizar E, Kralikova M, Mobarec JC, Shi L, Filizola M, et al Dopamine D2 receptors form higher order oligomers at physiological expression levels. The EMBO journal. 2008;27(17):2293–304. Epub 2008/08/01. doi: 10.1038/emboj.2008.153 ; PubMed Central PMCID: PMC2529367. 18668123 10.1038/emboj.2008.153 PMC2529367

[17] Bowman GR, Pande VS, Noé F. An Introduction to Markov State Models and Their Application to Long Timescale Molecular Simulation: Springer Science & Business Media; 2013.

[18] Prinz JH, Wu H, Sarich M, Keller B, Senne M, Held M, et al Markov models of molecular kinetics: Generation and validation. Journal of Chemical Physics. 2011;134(17). doi: 10.1063/1.3565032. WOS:000290393200011. 21548671 10.1063/1.3565032

[19] Laskowski RA, Swindells MB. LigPlot+: multiple ligand-protein interaction diagrams for drug discovery. J Chem Inf Model. 2011;51(10):2778–86. doi: 10.1021/ci200227u . 21919503 10.1021/ci200227u

[20] Michino M, Boateng CA, Donthamsetti P, Yano H, Bakare OM, Bonifazi A, et al Toward Understanding the Structural Basis of Partial Agonism at the Dopamine D3 Receptor. Journal of Medicinal Chemistry. 2017;60(2):580–93. doi: 10.1021/acs.jmedchem.6b01148 27983845 10.1021/acs.jmedchem.6b01148 PMC5563258

[21] Newman AH, Beuming T, Banala AK, Donthamsett P, Pongetti K, LaBounty A, et al Molecular Determinants of Selectivity and Efficacy at the Dopamine D3 Receptor. Journal of Medicinal Chemistry. 2012;55(15):6689–99. doi: 10.1021/jm300482h. WOS:000307264100004. 22632094 10.1021/jm300482h PMC3415572

[22] Stolzenberg S, Michino M, LeVine MV, Weinstein H, Shi L. Computational approaches to detect allosteric pathways in transmembrane molecular machines. Biochim Biophys Acta. 2016;1858(7 Pt B):1652–62. doi: 10.1016/j.bbamem.2016.01.010 ; PubMed Central PMCID: PMCPMC4877268. 26806157 10.1016/j.bbamem.2016.01.010 PMC4877268

[23] Michino M, Donthamsetti P, Beuming T, Banala A, Duan LH, Roux T, et al A Single Glycine in Extracellular Loop 1 Is the Critical Determinant for Pharmacological Specificity of Dopamine D2 and D3 Receptors. Molecular Pharmacology. 2013;84(6):854–64. doi: 10.1124/mol.113.087833. WOS:000326687200007. 24061855 10.1124/mol.113.087833 PMC3834143

[24] Bonifazi A, Yano H, Ellenberger MP, Muller L, Kumar V, Zou MF, et al Novel Bivalent Ligands Based on the Sumanirole Pharmacophore Reveal Dopamine D2 Receptor (D2R) Biased Agonism. J Med Chem. 2017;60(7):2890–907. doi: 10.1021/acs.jmedchem.6b01875 . 28300398 10.1021/acs.jmedchem.6b01875 PMC7594663

[25] Weichert D, Banerjee A, Hiller C, Kling RC, Hübner H, Gmeiner P. Molecular Determinants of Biased Agonism at the Dopamine D2 Receptor. Journal of Medicinal Chemistry. 2015;58(6):2703–17. doi: 10.1021/jm501889t 25734236 10.1021/jm501889t

[26] Conn PJ, Christopoulos A, Lindsley CW. Allosteric modulators of GPCRs: a novel approach for the treatment of CNS disorders. Nat Rev Drug Discov. 2009;8(1):41–54. doi: 10.1038/nrd2760 ; PubMed Central PMCID: PMCPMC2907734. 19116626 10.1038/nrd2760 PMC2907734

[27] Kountz TS, Lee KS, Aggarwal-Howarth S, Curran E, Park JM, Harris DA, et al Endogenous N-terminal Domain Cleavage Modulates alpha1D-Adrenergic Receptor Pharmacodynamics. J Biol Chem. 2016;291(35):18210–21. doi: 10.1074/jbc.M116.729517 ; PubMed Central PMCID: PMCPMC5000069. 27382054 10.1074/jbc.M116.729517 PMC5000069

[28] Belmer A, Doly S, Setola V, Banas SM, Moutkine I, Boutourlinsky K, et al Role of the N-terminal region in G protein-coupled receptor functions: negative modulation revealed by 5-HT2B receptor polymorphisms. Mol Pharmacol. 2014;85(1):127–38. doi: 10.1124/mol.113.089086 . 24174497 10.1124/mol.113.089086

[29] Knapman A, Santiago M, Connor M. Buprenorphine signalling is compromised at the N40D polymorphism of the human mu opioid receptor in vitro. Br J Pharmacol. 2014;171(18):4273–88. doi: 10.1111/bph.12785 ; PubMed Central PMCID: PMCPMC4241093. 24846673 10.1111/bph.12785 PMC4241093

[30] Knapman A, Santiago M, Connor M. A6V polymorphism of the human mu-opioid receptor decreases signalling of morphine and endogenous opioids in vitro. Br J Pharmacol. 2015;172(9):2258–72. doi: 10.1111/bph.13047 ; PubMed Central PMCID: PMCPMC4403092. 25521224 10.1111/bph.13047 PMC4403092

[31] Chien EY, Liu W, Zhao Q, Katritch V, Han GW, Hanson MA, et al Structure of the human dopamine D3 receptor in complex with a D2/D3 selective antagonist. Science. 2010;330(6007):1091–5. doi: 10.1126/science.1197410 ; PubMed Central PMCID: PMCPMC3058422. 21097933 10.1126/science.1197410 PMC3058422

[32] Sherman W, Day T, Jacobson MP, Friesner RA, Farid R. Novel procedure for modeling ligand/receptor induced fit effects. J Med Chem. 2006;49(2):534–53. doi: 10.1021/jm050540c . 16420040 10.1021/jm050540c

[33] Michino M, Free RB, Doyle TB, Sibley DR, Shi L. Structural basis for Na(+)-sensitivity in dopamine D2 and D3 receptors. Chem Commun (Camb). 2015;51(41):8618–21. doi: 10.1039/c5cc02204e . 25896577 10.1039/c5cc02204e PMC5234269

[34] MacKerell AD, Bashford D, Bellott M, Dunbrack RL, Evanseck JD, Field MJ, et al All-atom empirical potential for molecular modeling and dynamics studies of proteins. J Phys Chem B. 1998;102(18):3586–616. doi: 10.1021/jp973084f . 24889800 10.1021/jp973084f

[35] Mackerell AD Jr., Feig M, Brooks CL, 3rd. Extending the treatment of backbone energetics in protein force fields: limitations of gas-phase quantum mechanics in reproducing protein conformational distributions in molecular dynamics simulations. J Comput Chem. 2004;25(11):1400–15. doi: 10.1002/jcc.20065 . 15185334 10.1002/jcc.20065

[36] Best RB, Zhu X, Shim J, Lopes PE, Mittal J, Feig M, et al Optimization of the additive CHARMM all-atom protein force field targeting improved sampling of the backbone phi, psi and side-chain chi(1) and chi(2) dihedral angles. J Chem Theory Comput. 2012;8(9):3257–73. doi: 10.1021/ct300400x ; PubMed Central PMCID: PMCPMC3549273. 23341755 10.1021/ct300400x PMC3549273

[37] Klauda JB, Venable RM, Freites JA, O'Connor JW, Tobias DJ, Mondragon-Ramirez C, et al Update of the CHARMM all-atom additive force field for lipids: validation on six lipid types. J Phys Chem B. 2010;114(23):7830–43. doi: 10.1021/jp101759q ; PubMed Central PMCID: PMCPMC2922408. 20496934 10.1021/jp101759q PMC2922408

[38] Huang L, Roux B. Automated Force Field Parameterization for Non-Polarizable and Polarizable Atomic Models Based on Ab Initio Target Data. J Chem Theory Comput. 2013;9(8). doi: 10.1021/ct4003477 ; PubMed Central PMCID: PMCPMC3819940. 24223528 10.1021/ct4003477 PMC3819940

[39] Vanommeslaeghe K, Hatcher E, Acharya C, Kundu S, Zhong S, Shim J, et al CHARMM general force field: A force field for drug-like molecules compatible with the CHARMM all-atom additive biological force fields. J Comput Chem. 2010;31(4):671–90. doi: 10.1002/jcc.21367 ; PubMed Central PMCID: PMCPMC2888302. 19575467 10.1002/jcc.21367 PMC2888302

[40] Feller SE, Zhang Y, Pastor RW, Brooks BR. Constant pressure molecular dynamics simulation: The Langevin piston method. J Chem Phys. 1995;103(11):4613–21.

[41] Scherer MK, Trendelkamp-Schroer B, Paul F, Perez-Hernandez G, Hoffmann M, Plattner N, et al PyEMMA 2: A Software Package for Estimation, Validation, and Analysis of Markov Models. J Chem Theory Comput. 2015;11(11):5525–42. doi: 10.1021/acs.jctc.5b00743 . 26574340 10.1021/acs.jctc.5b00743

[42] Perez-Hernandez G, Paul F, Giorgino T, De Fabritiis G, Noe F. Identification of slow molecular order parameters for Markov model construction. J Chem Phys. 2013;139(1):015102 doi: 10.1063/1.4811489 . 23822324 10.1063/1.4811489

[43] Schwantes CR, Pande VS. Improvements in Markov State Model Construction Reveal Many Non-Native Interactions in the Folding of NTL9. J Chem Theory Comput. 2013;9(4):2000–9. doi: 10.1021/ct300878a ; PubMed Central PMCID: PMCPMC3673732. 23750122 10.1021/ct300878a PMC3673732

[44] Noé F, Nüske F. A Variational Approach to Modeling Slow Processes in Stochastic Dynamical Systems. Multiscale Modeling & Simulation. 2013;11(2):635–55. doi: 10.1137/110858616

[45] Nuske F, Keller BG, Perez-Hernandez G, Mey AS, Noe F. Variational Approach to Molecular Kinetics. J Chem Theory Comput. 2014;10(4):1739–52. doi: 10.1021/ct4009156 . 26580382 10.1021/ct4009156

[46] McGibbon RT, Pande VS. Variational cross-validation of slow dynamical modes in molecular kinetics. J Chem Phys. 2015;142(12):124105 doi: 10.1063/1.4916292 ; PubMed Central PMCID: PMCPMC4398134. 25833563 10.1063/1.4916292 PMC4398134

[47] Abramyan AM, Stolzenberg S, Li Z, Loland CJ, Noe F, Shi L. The Isomeric Preference of an Atypical Dopamine Transporter Inhibitor Contributes to Its Selection of the Transporter Conformation. ACS Chem Neurosci. 2017 doi: 10.1021/acschemneuro.7b00094 . 28441487 10.1021/acschemneuro.7b00094 PMC11931626

[48] Trendelkamp-Schroer B, Noe F. Efficient Bayesian estimation of Markov model transition matrices with given stationary distribution. J Chem Phys. 2013;138(16):164113 doi: 10.1063/1.4801325 . 23635117 10.1063/1.4801325

[49] Roblitz S, Weber M. Fuzzy spectral clustering by PCCA plus: application to Markov state models and data classification. Advances in Data Analysis and Classification. 2013;7(2):147–79. doi: 10.1007/s11634-013-0134-6. WOS:000320331800003.

[50] Singhal N, Pande VS. Error analysis and efficient sampling in Markovian state models for molecular dynamics. J Chem Phys. 2005;123(20):204909 doi: 10.1063/1.2116947 . 16351319 10.1063/1.2116947

