# Supplementary material for: The E2.65A mutation disrupts dynamic binding poses of SB269652 at the dopamine D2 and D3 receptors
Source: PLoS Comput Biol. 2018 Jan 16;14(1):e1005948. doi: 10.1371/journal.pcbi.1005948 (PMC5786319; doi:10.1371/journal.pcbi.1005948)
Supplement: S1 Fig — The tetrahydroisoquinoline (PP) and indole-2-carboxamide moiety (SP) of SB269652 are shown in green and pink respectively. COM5 and COM6 refer to the centers of mass of the 5- and 6-member rings of the SP, respectively. The input features for MSM analysis include 12 distances from the nitrogen atoms of SB269652 to the indicated receptor Cβ atoms, 2 intramolecular distances of SB269652, and the projections of the vectors from COM5 or COM6 to N4 along the axis perpendicular to membrane (see Methods). (PDF) [file pcbi.1005948.s001.pdf]

S1 Fig. Chemical structure of SB269652 and the input features for MSM analysis. The tetrahydroisoquinoline (PP) and indole-2-carboxamide moiety (SP) of SB269652 are shown in green and pink respectively. COM5 and COM6 refer to the centers of mass of the 5- and 6-member rings of the SP, respectively. The input features for MSM analysis include 12 distances from the nitrogen atoms of SB269652 to the indicated receptor Cβ atoms, 2 intramolecular distances of SB269652, and the projections of the vectors from COM5 or COM6 to N4 along the axis perpendicular to membrane (see Methods).

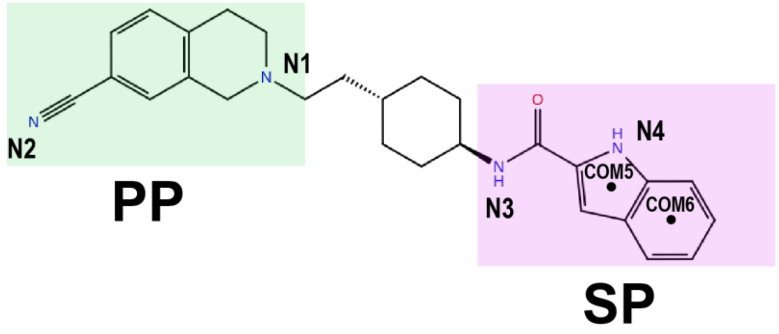

Index	Atom name	SB269652 atom
**1**	$Asp^{3.32}$ Cβ	N1
**2**	$Trp^{7.39}$ Cβ	N1
**3**	$Leu^{3.33}$ Cβ	N2
**4**	$Phe^{5.38}$ Cβ	N2
**5**	$Val^{5.39}$ Cβ	N2
**6**	$Ser^{5.42}$ Cβ	N2
**7**	$Ser^{5.43}$ Cβ	N2
**8**	$Ser^{5.46}$ Cβ	N2
**9**	$Val^{2.61}$ Cβ	N3
**10**	$Leu^{2.64}$ Cβ	N3
**11**	$Ser^{7.36}$ Cβ	N3
**12**	$Leu^{2.64}$ Cβ	N4
**13**	SB269652 N4	N3
**14**	SB269652 N4	O
**15**	SB269652 N4	COM5
**16**	SB269652 N4	COM6
