# Supplementary material for: The E2.65A mutation disrupts dynamic binding poses of SB269652 at the dopamine D2 and D3 receptors
Source: PLoS Comput Biol. 2018 Jan 16;14(1):e1005948. doi: 10.1371/journal.pcbi.1005948 (PMC5786319; doi:10.1371/journal.pcbi.1005948)

**S2 Fig. The salt bridge between the N1 atom of SB269652 and Asp$^{3.32}$ remains stable in all conditions.** Distributions of the minimum distances between the N1 nitrogen atom of SB269652 and carboxyl oxygen atoms of Asp$^{3.32}$ for each MS of the indicated conditions are shown. The MSs for each condition are colored according to Fig 1.

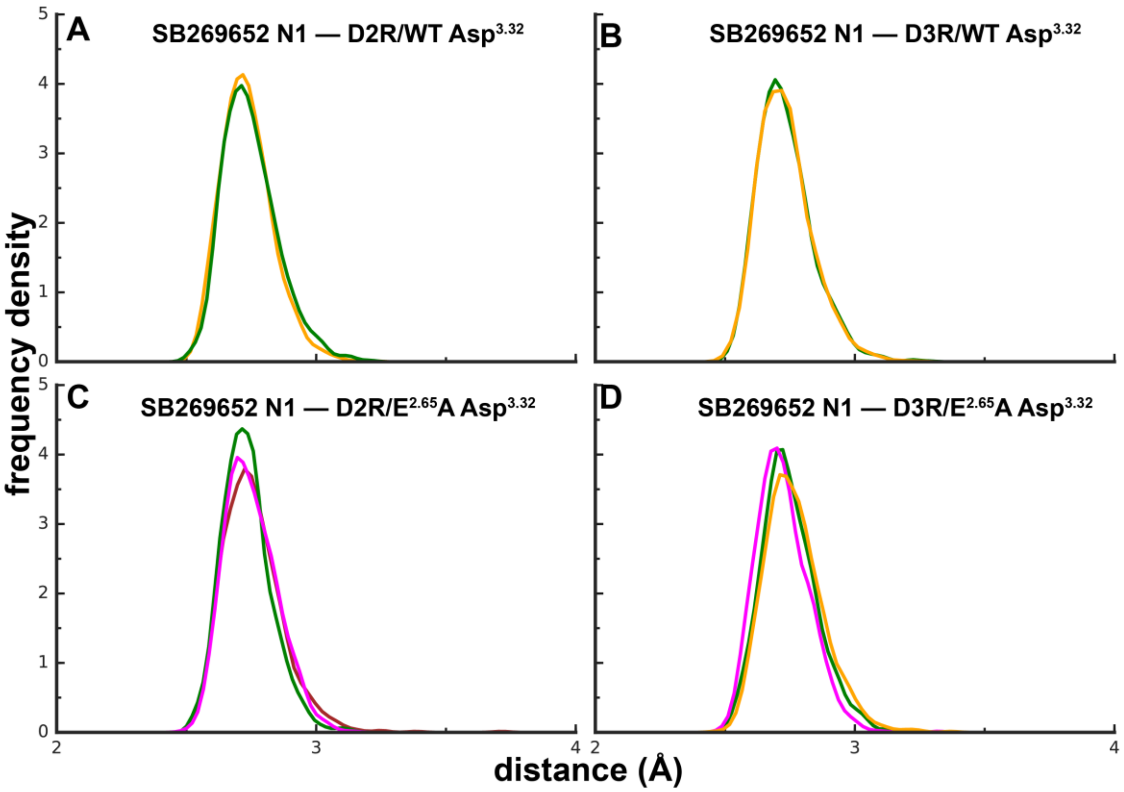

Supplement: S2 Fig — Distributions of the minimum distances between the N1 nitrogen atom of SB269652 and carboxyl oxygen atoms of Asp3.32 for each MS of the indicated conditions are shown. The MSs for each condition are colored according to Fig 1. (PDF) [file pcbi.1005948.s002.pdf]
