# Supplementary material for: The E2.65A mutation disrupts dynamic binding poses of SB269652 at the dopamine D2 and D3 receptors
Source: PLoS Comput Biol. 2018 Jan 16;14(1):e1005948. doi: 10.1371/journal.pcbi.1005948 (PMC5786319; doi:10.1371/journal.pcbi.1005948)

S3 Fig. Distribution of the distances from the N3 and N4 atoms of SB269652 to the Cβ atom of Ala$^{2.65}$ in mutant receptors. The MSs for each condition are colored according to Fig 1.

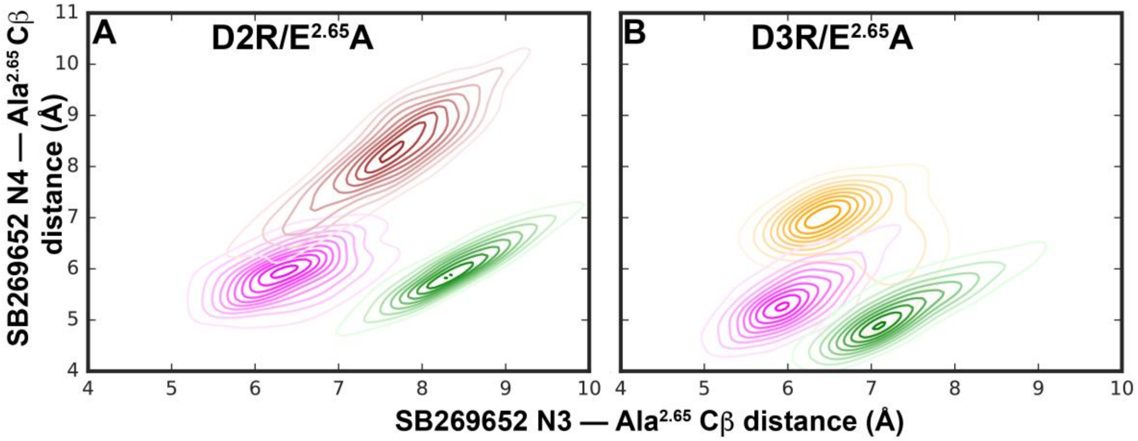

Supplement: S3 Fig — The MSs for each condition are colored according to Fig 1. (PDF) [file pcbi.1005948.s003.pdf]
