# Supplementary material for: The E2.65A mutation disrupts dynamic binding poses of SB269652 at the dopamine D2 and D3 receptors
Source: PLoS Comput Biol. 2018 Jan 16;14(1):e1005948. doi: 10.1371/journal.pcbi.1005948 (PMC5786319; doi:10.1371/journal.pcbi.1005948)
Supplement: S4 Fig — The secondary structures were classified by DSSP v2.0.4 [1]. The boundary between NT and TM1 is indicated by the dotted line. (PDF) [file pcbi.1005948.s004.pdf]

S4 Fig. Distribution of secondary structure content for each of the N terminal residues. The secondary structures were classified by DSSP v2.0.4 [1]. The boundary between NT and TM1 is indicated by the dotted line.

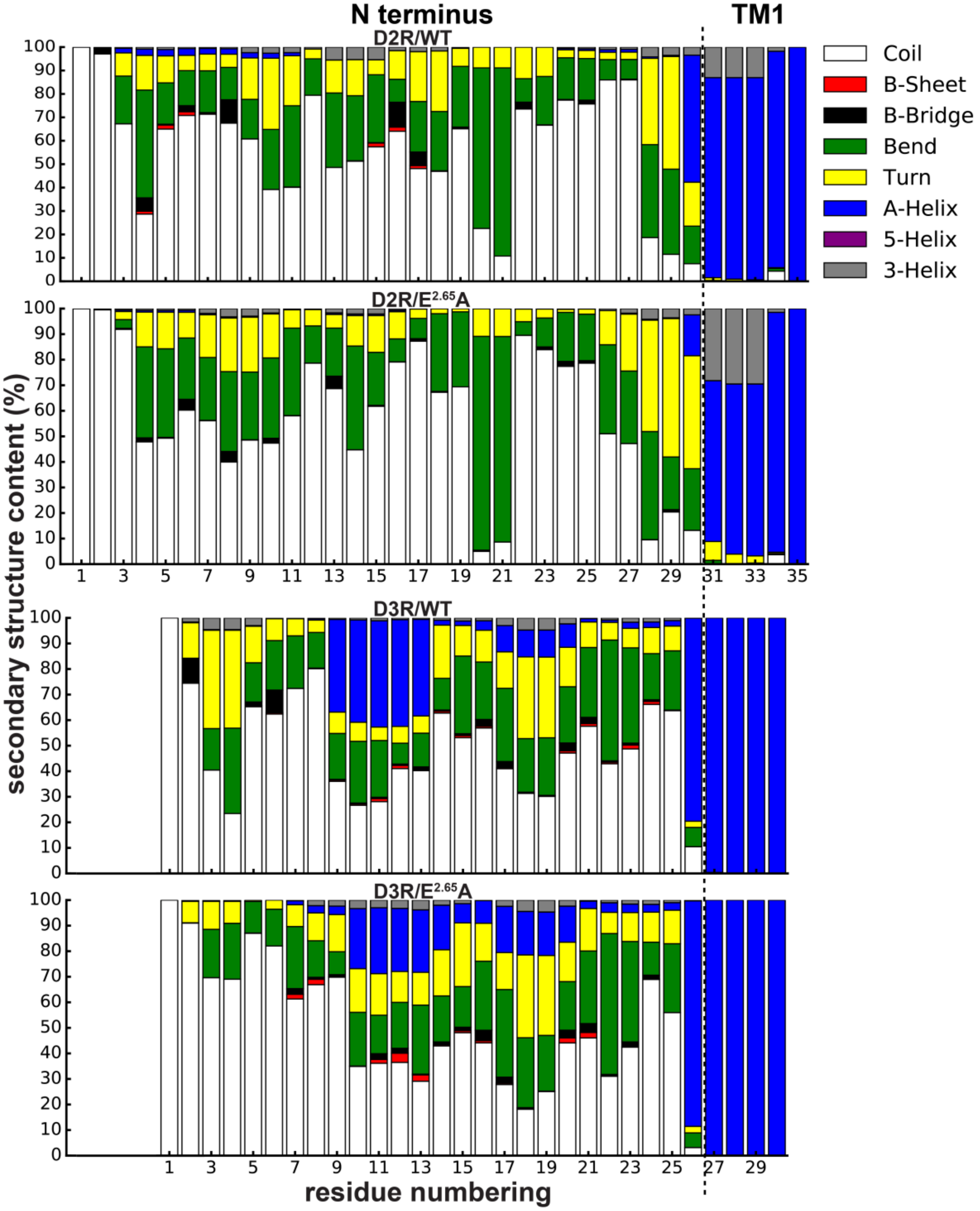

Reference

1. Kabsch W, Sander C. Dictionary of protein secondary structure: pattern recognition of hydrogen-bonded and geometrical features. Biopolymers. 1983;22(12):2577-637. doi: 10.1002/bip.360221211. PubMed PMID: 6667333.
