# Supplementary material for: The E2.65A mutation disrupts dynamic binding poses of SB269652 at the dopamine D2 and D3 receptors
Source: PLoS Comput Biol. 2018 Jan 16;14(1):e1005948. doi: 10.1371/journal.pcbi.1005948 (PMC5786319; doi:10.1371/journal.pcbi.1005948)
Supplement: S5 Fig — The NT is within 5 Å (heavy atom-heavy atom distance) of the small-molecule ligands that at least partially occupy the OBS in the crystal structures of (A) Chemokine receptors CCR2 (PDB: 5T1A) [1] and (B) CXCR4 (PDB: 3ODU) [2], (C) lysophospholipid receptor-1 (PDB: 4Z34) [3], (D) μ-opioid receptor (PDB: 5C1M) [4], (E) sphingosine 1-phosphate receptor subtype 1(PDB: 3V2Y) [5], and (F) cannabinoid receptor CB1 (PDB: 5TGZ, 5U09, 5XR8, and 5XRA) [6–8]. (PDF) [file pcbi.1005948.s005.pdf]

S5 Fig. The high-resolution crystal structures of class-A GPCRs with the NT in contact with small-molecule ligands. The NT is within 5 Å (heavy atom-heavy atom distance) of the small-molecule ligands that at least partially occupy the OBS in the crystal structures of (**A**) Chemokine receptors CCR2 (PDB: 5T1A) [1] and (**B**) CXCR4 (PDB: 3ODU) [2], (**C**) lysophospholipid receptor-1 (PDB: 4Z34) [3]**,** (**D**) μ-opioid receptor (PDB: 5C1M) [4], (**E**) sphingosine 1-phosphate receptor subtype 1(PDB: 3V2Y) [5], and (**F**) cannabinoid receptor CB1 (PDB: 5TGZ, 5U09, 5XR8, and 5XRA) [6-8]**.**

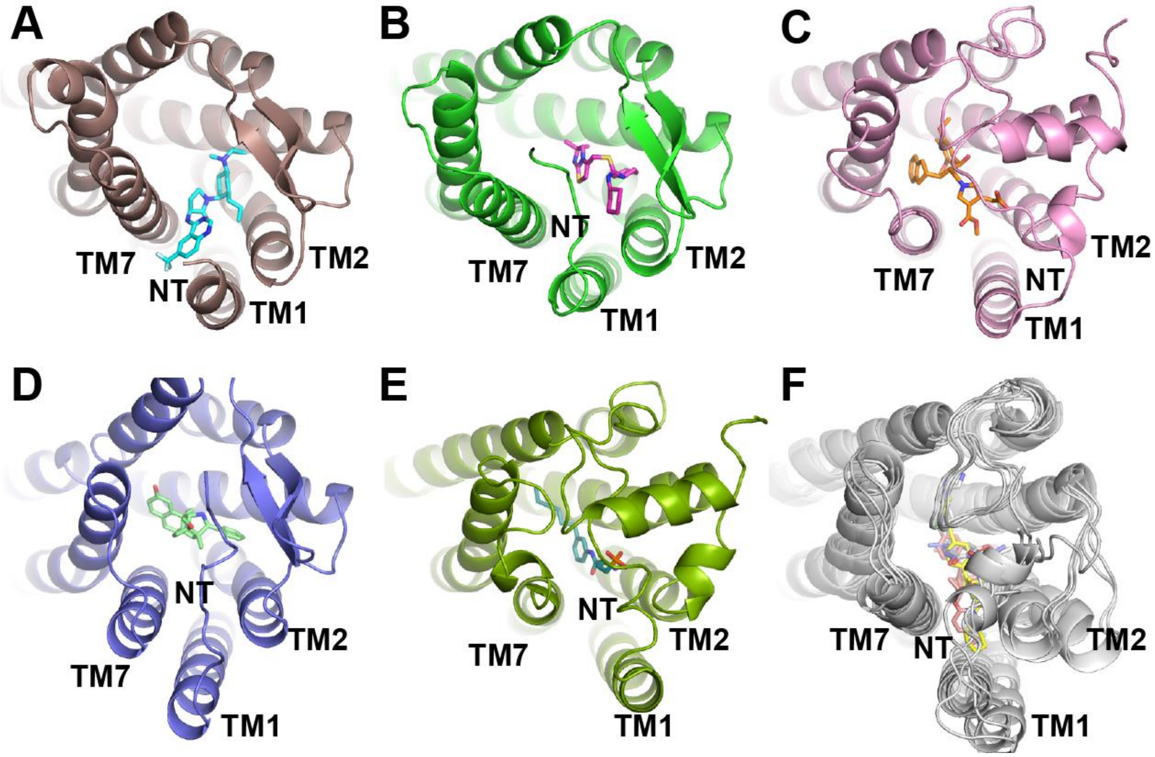

References

1. Zheng Y, Qin L, Zacarias NV, de Vries H, Han GW, Gustavsson M, et al. Structure of CC chemokine receptor 2 with orthosteric and allosteric antagonists. Nature. 2016;540(7633):458-61. doi: 10.1038/nature20605. PubMed PMID: 27926736; PubMed Central PMCID: PMCPMC5159191.

2. Wu B, Chien EY, Mol CD, Fenalti G, Liu W, Katritch V, et al. Structures of the CXCR4 chemokine GPCR with small-molecule and cyclic peptide antagonists. Science. 2010;330(6007):1066-71. doi: 10.1126/science.1194396. PubMed PMID: 20929726; PubMed Central PMCID: PMCPMC3074590.

3. Chrencik JE, Roth CB, Terakado M, Kurata H, Omi R, Kihara Y, et al. Crystal Structure of Antagonist Bound Human Lysophosphatidic Acid Receptor 1. Cell. 2015;161(7):1633-43. doi: 10.1016/j.cell.2015.06.002. PubMed PMID: 26091040; PubMed Central PMCID: PMCPMC4476059.

4.Huang W, Manglik A, Venkatakrishnan AJ, Laeremans T, Feinberg EN, Sanborn AL, et al. Structural insights into μ-opioid receptor activation. Nature. 2015;524(7565):315-21. doi: 10.1038/nature14886. PubMed PMID: 26245379; PubMed Central PMCID: PMCPMC4639397.

5. Hanson MA, Roth CB, Jo E, Griffith MT, Scott FL, Reinhart G, et al. Crystal structure of a lipid G protein-coupled receptor. Science. 2012;335(6070):851-5. doi: 10.1126/science.1215904. PubMed PMID: 22344443; PubMed Central PMCID: PMCPMC3338336.
6. Hua T, Vemuri K, Pu M, Qu L, Han GW, Wu Y, et al. Crystal Structure of the Human Cannabinoid Receptor CB1. Cell. 2016;167(3):750-62 e14. doi: 10.1016/j.cell.2016.10.004. PubMed PMID: 27768894; PubMed Central PMCID: PMCPMC5322940.
7. Shao Z, Yin J, Chapman K, Grzemska M, Clark L, Wang J, et al. High-resolution crystal structure of the human CB1 cannabinoid receptor. Nature. 2016. doi: 10.1038/nature20613. PubMed PMID: 27851727; PubMed Central PMCID: PMCPMC5433929.
8. Hua T, Vemuri K, Nikas SP, Laprairie RB, Wu Y, Qu L, et al. Crystal structures of agonist-bound human cannabinoid receptor CB1. Nature. 2017;547(7664):468-71. doi: 10.1038/nature23272. PubMed PMID: 28678776.
