# Supplementary material for: The E2.65A mutation disrupts dynamic binding poses of SB269652 at the dopamine D2 and D3 receptors
Source: PLoS Comput Biol. 2018 Jan 16;14(1):e1005948. doi: 10.1371/journal.pcbi.1005948 (PMC5786319; doi:10.1371/journal.pcbi.1005948)

S6 Fig. Implied time scales for 50- and 75-microstate MSMs. Implied timescales (ITS) are plotted against lag time. The ITS of the maximum likelihood Bayesian Markov model are shown in solid lines, whereas the means and the 95% confidence intervals (computed by Bayesian sampling) are shown in dashed and shaded areas, respectively. In blue, red, and green, … are the 1st, 2nd, and 3rd, … slowest ITS.

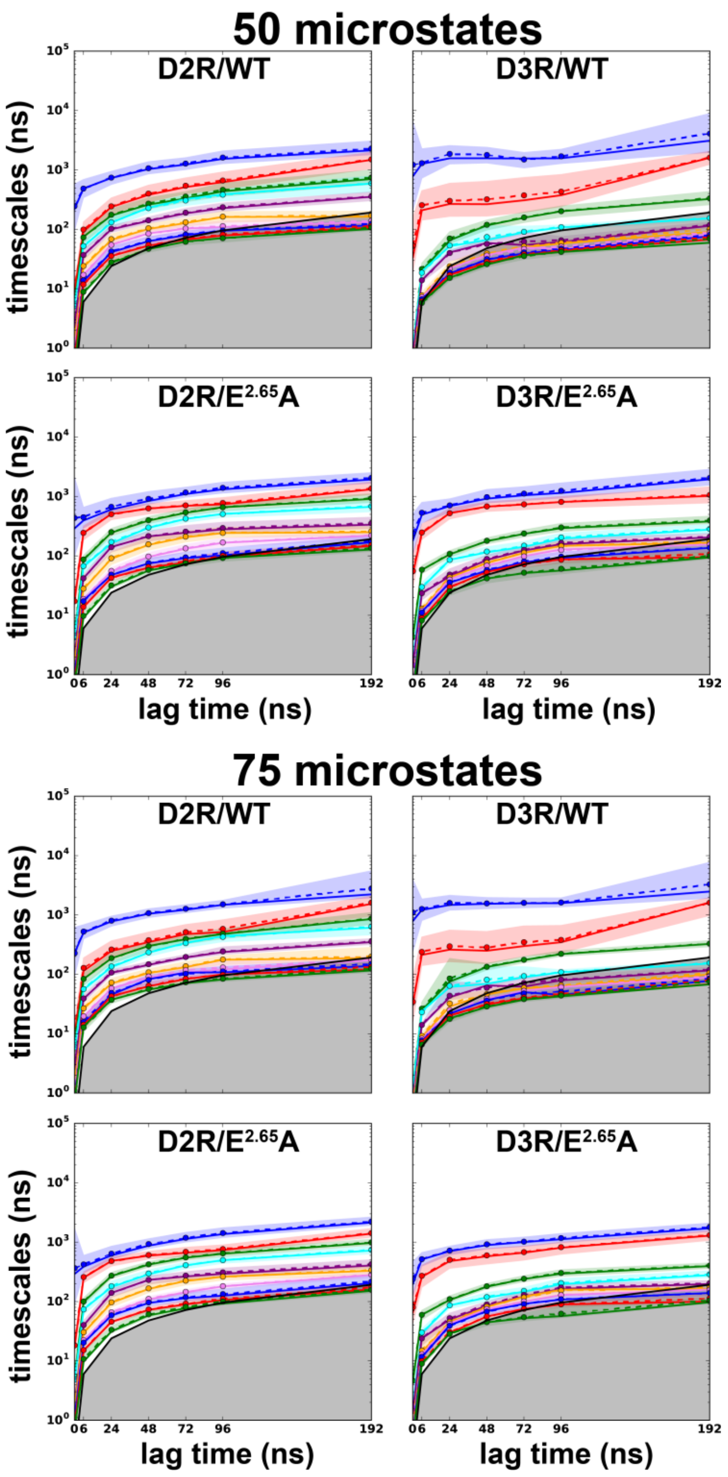

Supplement: S6 Fig — Implied timescales (ITS) are plotted against lag time. The ITS of the maximum likelihood Bayesian Markov model are shown in solid lines, whereas the means and the 95% confidence intervals (computed by Bayesian sampling) are shown in dashed and shaded areas, respectively. In blue, red, and green, … are the 1st, 2nd, and 3rd, … slowest ITS. (PDF) [file pcbi.1005948.s006.pdf]
