# Supplementary figures and images for: The E2.65A mutation disrupts dynamic binding poses of SB269652 at the dopamine D2 and D3 receptors
Source: PLoS Comput Biol. 2018 Jan 16;14(1):e1005948. doi: 10.1371/journal.pcbi.1005948 (PMC5786319; doi:10.1371/journal.pcbi.1005948)

**S7 Fig. ITS for 100- and 150-microstate MSMs.** The color coding is the same as in S6 Fig.

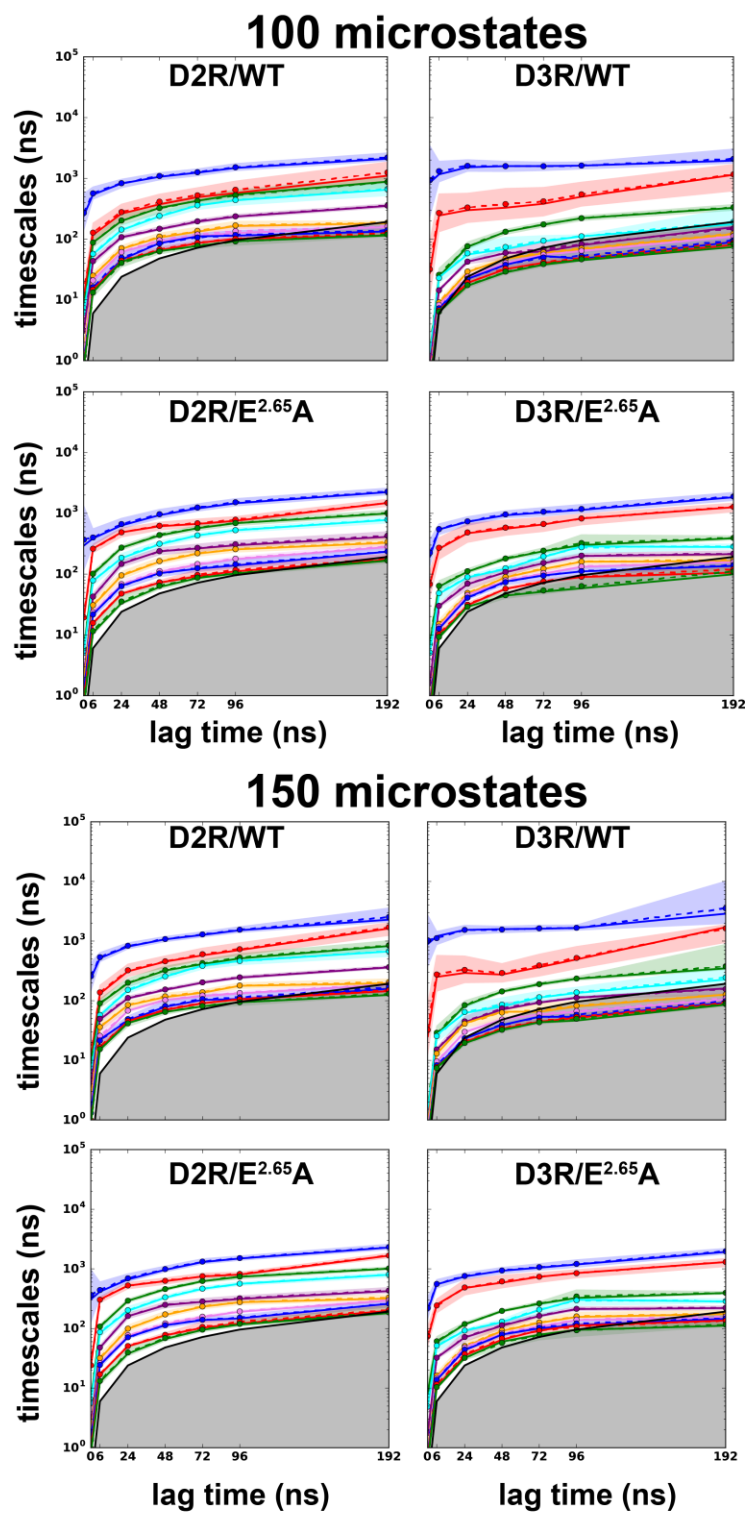

Supplement: S7 Fig — The color coding is the same as in S6 Fig. (PDF) [file pcbi.1005948.s007.pdf]

**S8 Fig. ITS for 200- and 300-microstate MSMs.** The color coding is the same as in S6 Fig.

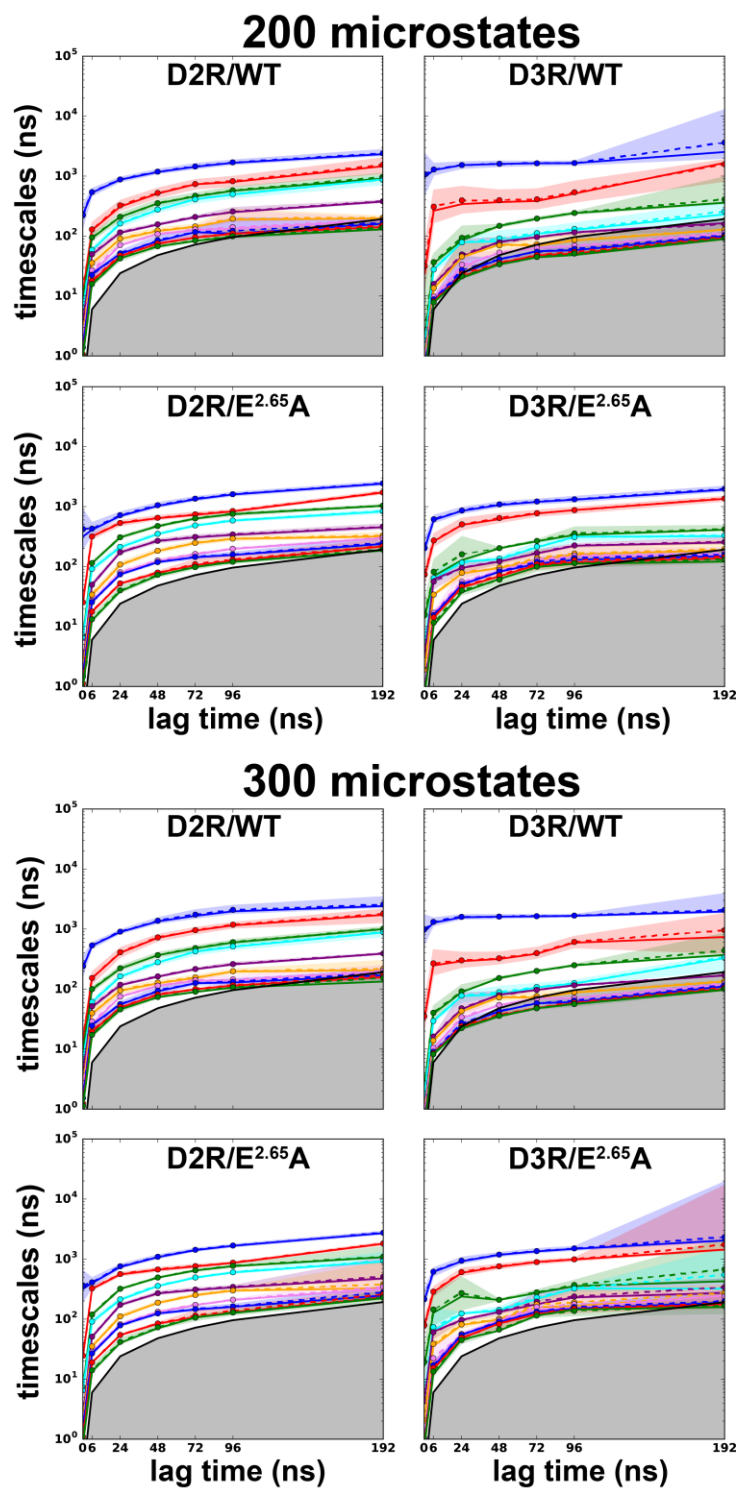

Supplement: S8 Fig — The color coding is the same as in S6 Fig. (PDF) [file pcbi.1005948.s008.pdf]

**S10 Fig. Chapman-Kolmogorov test for D3R/WT and D3R/E<sup>2.65</sup>A.** See S9 Fig for details.

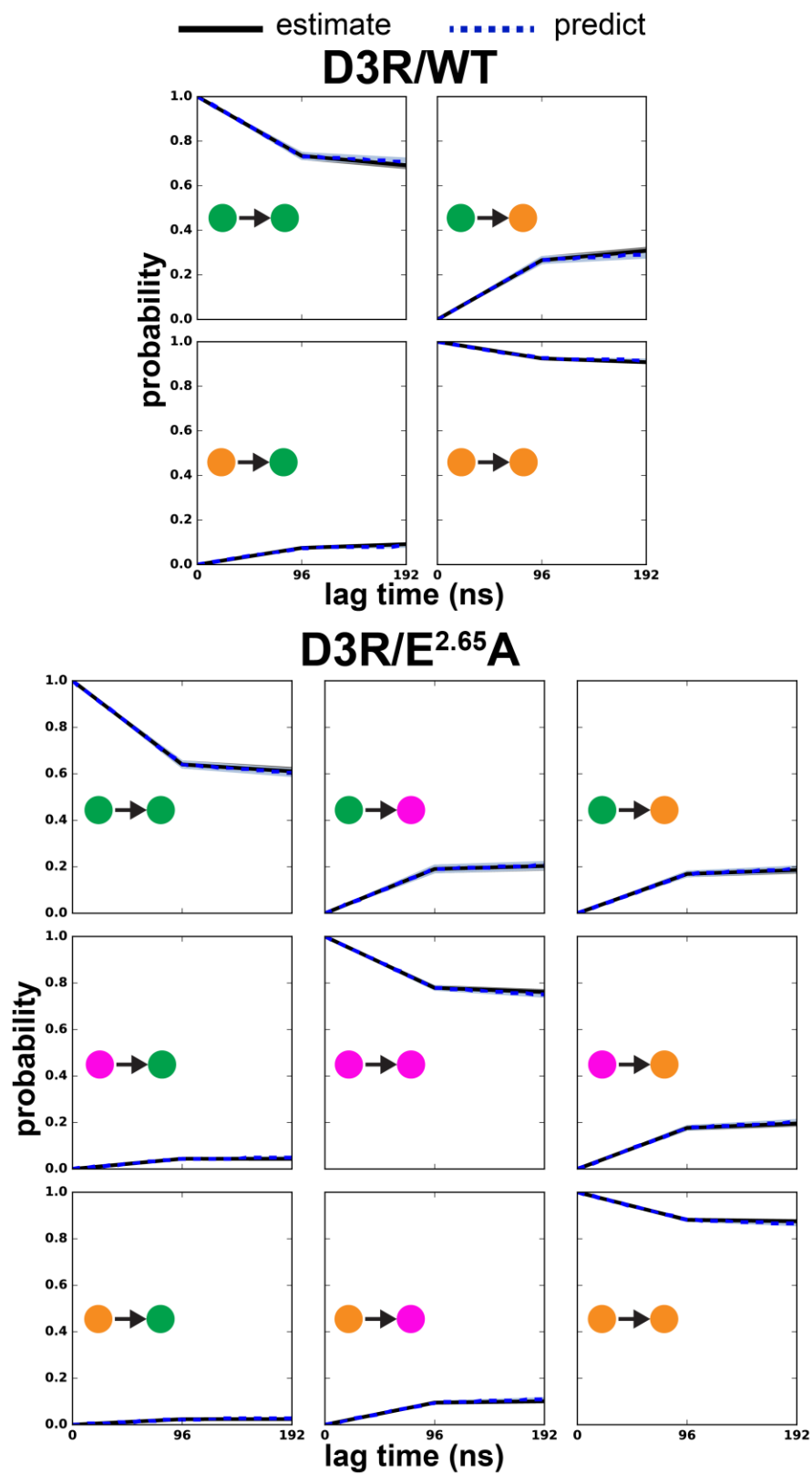

Supplement: S10 Fig — See S9 Fig for details. (PDF) [file pcbi.1005948.s010.pdf]
