# Supplementary material for: The E2.65A mutation disrupts dynamic binding poses of SB269652 at the dopamine D2 and D3 receptors
Source: PLoS Comput Biol. 2018 Jan 16;14(1):e1005948. doi: 10.1371/journal.pcbi.1005948 (PMC5786319; doi:10.1371/journal.pcbi.1005948)

S9 Fig. Chapman-Kolmogorov test for D2R/WT and D2R/E$^{2.65}$A. For each of the MSs in our models we initialize the population in that state and compare the evolution of the population predicted from the MSM at given lag times (dashed blue lines) to that measured on the trajectories (black lines) at the same lag times. The shaded areas (light blue) represent 95% confidence intervals computed by bootstrapping. The colors of the MSs are the same as in Fig 1.

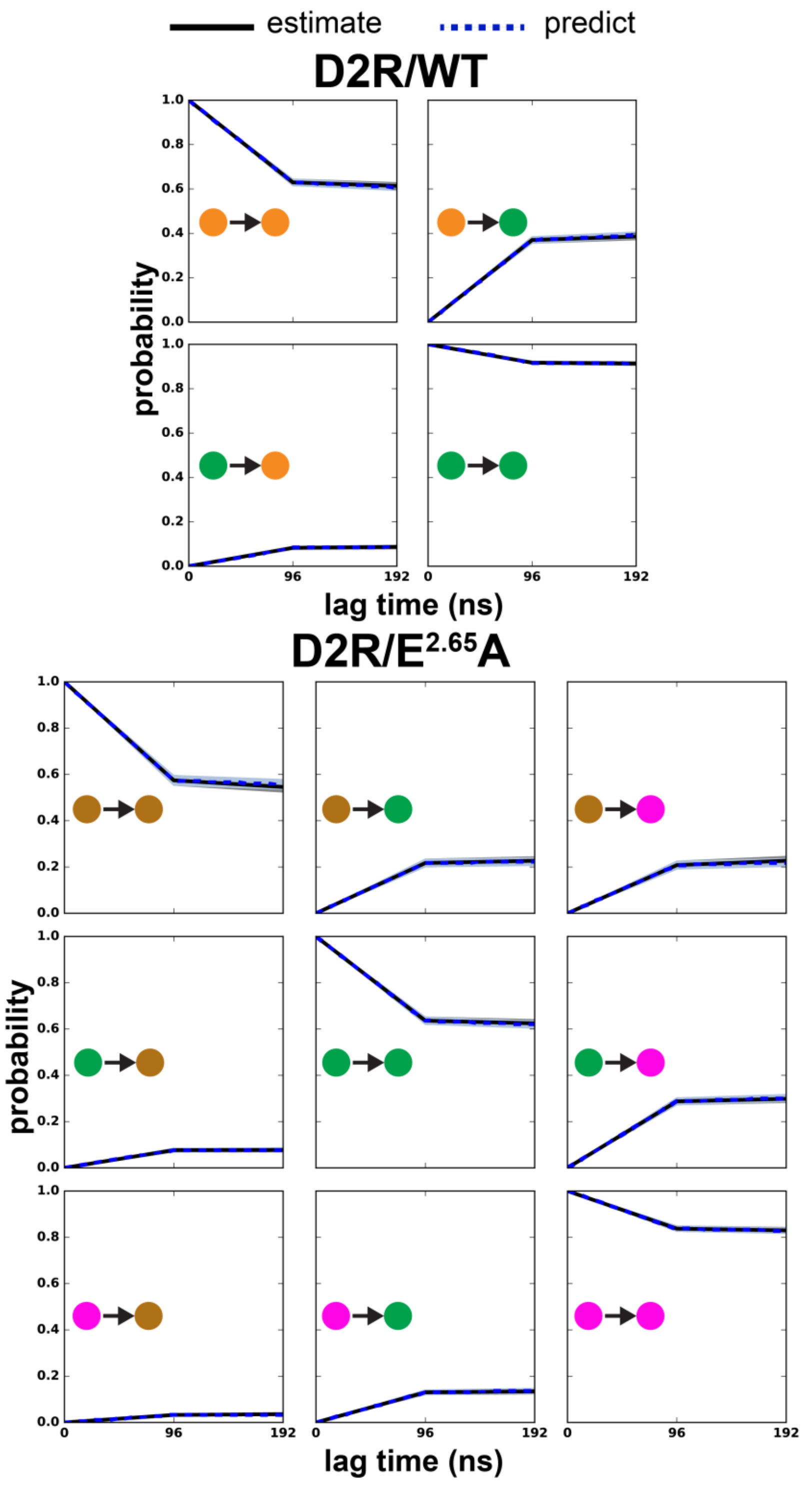

Supplement: S9 Fig — For each of the MSs in our models we initialize the population in that state and compare the evolution of the population predicted from the MSM at given lag times (dashed blue lines) to that measured on the trajectories (black lines) at the same lag times. The shaded areas (light blue) represent 95% confidence intervals computed by bootstrapping. The colors of the MSs are the same as in Fig 1. (PDF) [file pcbi.1005948.s009.pdf]
