# Supplementary material for: The E2.65A mutation disrupts dynamic binding poses of SB269652 at the dopamine D2 and D3 receptors
Source: PLoS Comput Biol. 2018 Jan 16;14(1):e1005948. doi: 10.1371/journal.pcbi.1005948 (PMC5786319; doi:10.1371/journal.pcbi.1005948)
Supplement: S1 Table — The means and standard deviations of the interaction frequencies for each residue were calculated using 3 sets of 500 MSM Bayesian samples, each set containing 1500 frames per MS. Residues having contact frequencies above 25% in at least one of the MS are listed and the contact frequencies above 25% are in bold. OBS and SBP residues are highlighted in cyan and yellow, respectively. (PDF) [file pcbi.1005948.s011.pdf]

S1 Table. SB269652 interacting residues. The means and standard deviations of the interaction frequencies for each residue were calculated using 3 sets of 500 MSM Bayesian samples, each set containing 1500 frames per MS. Residues having contact frequencies above 25% in at least one of the MS are listed and the contact frequencies above 25% are in bold. The OBS and SBP residues identified in [7] are highlighted in cyan and yellow, respectively.

Index	D2R/WT			D3R/WT		
	Residue	green (%)	orange (%)	Residue	green (%)	orange (%)
NT	MET1	**39.3 ± 1.0**	**25.7 ± 1.7**			
	TRP18	**25.0 ± 1.6**	11.4 ± 0.7	LEU7	**60.7 ± 0.9**	**51.9 ± 0.8**
	PHE22	**33.9 ± 0.6**	23.5 ± 1.3	LEU11	**38.2 ± 2.3**	**31.5 ± 1.3**
	ASN23	**25.2 ± 0.8**	20.0 ± 0.4	TYR13	21.4 ± 0.6	**32.7 ± 1.9**
1.32	TYR34	**28.6 ± 0.2**	6.6 ± 0.8	HIS29	**29.5 ± 2.1**	**45.5 ± 1.4**
2.61	VAL91	**93.1 ± 0.2**	**84.2 ± 0.7**	VAL86	**94.9 ± 0.8**	**93.9 ± 1.3**
2.64	LEU94	**96.2 ± 0.3**	**87.8 ± 0.8**	LEU89	**100.0 ± 0.1**	**99.8 ± 0.1**
2.65	GLU95	**94.7 ± 0.2**	**91.4 ± 0.5**	GLU90	**99.5 ± 0.2**	**98.6 ± 0.2**
EL1	GLY98	**93.8 ± 0.4**	**76.2 ± 1.6**	GLY93	**99.4 ± 0.1**	**99.1 ± 0.3**
EL1	GLU99	**54.6 ± 0.7**	**39.5 ± 0.9**	GLY94	**99.4 ± 0.0**	**98.8 ± 0.3**
3.28	PHE110	**88.9 ± 0.6**	**80.4 ± 1.3**	PHE106	**77.0 ± 0.8**	**82.5 ± 0.8**
3.32	ASP114	**100.0 ± 0.0**	**100.0 ± 0.0**	ASP110	**100.0 ± 0.0**	**100.0 ± 0.0**
3.33	VAL115	**99.9 ± 0.1**	**100.0 ± 0.1**	VAL111	**99.9 ± 0.2**	**99.7 ± 0.1**
3.36	CYS118	**95.8 ± 0.2**	**99.0 ± 0.3**	CYS114	**83.4 ± 1.9**	**90.6 ± 0.8**
EL2.50	CYS182	**27.9 ± 0.2**	9.0 ± 0.2	CYS181	**33.3 ± 0.8**	**52.1 ± 0.5**
EL2.51	ILE183	**97.2 ± 0.3**	**99.7 ± 0.2**	SER182	**77.0 ± 0.6**	**72.7 ± 0.9**
EL2.52	ILE184	**27.5 ± 0.5**	0.6 ± 0.1	ILE183	**97.0 ± 0.4**	**96.0 ± 0.7**
5.38	PHE189	11.0 ± 0.4	4.9 ± 0.5	PHE188	**96.2 ± 0.6**	**95.8 ± 0.4**
5.39	VAL190	**93.5 ± 0.9**	**96.6 ± 0.4**	VAL189	**99.6 ± 0.1**	**99.6 ± 0.1**
5.42	SER193	**92.9 ± 0.3**	**99.8 ± 0.1**	SER192	**100.0 ± 0.1**	**99.9 ± 0.1**
5.43	SER194	**88.3 ± 0.3**	**99.7 ± 0.1**	SER193	14.1 ± 1.0	14.0 ± 0.5
5.46	SER197	**82.4 ± 0.9**	**97.5 ± 0.4**	SER196	15.6 ± 2.1	17.6 ± 0.9
6.48	TRP357	**100.0 ± 0.0**	**100.0 ± 0.0**	TRP342	**99.8 ± 0.2**	**99.8 ± 0.1**
6.51	PHE360	**100.0 ± 0.0**	**100.0 ± 0.0**	PHE345	**100.0 ± 0.0**	**100.0 ± 0.0**
6.52	PHE361	**99.8 ± 0.1**	**99.8 ± 0.2**	PHE346	**94.3 ± 0.9**	**95.1 ± 0.5**
6.55	HIS364	**80.4 ± 0.8**	**94.3 ± 0.3**	HIS349	**62.6 ± 1.5**	**43.0 ± 1.5**
7.32	PRO376	3.9 ± 0.4	16.9 ± 0.7	PRO362	**46.3 ± 0.7**	**74.2 ± 0.3**
7.33	VAL377	3.0 ± 0.4	14.4 ± 0.6	GLU363	**28.3 ± 0.5**	**46.2 ± 1.3**
7.35	TYR379	**29.4 ± 0.8**	**33.7 ± 0.8**	TYR365	**95.3 ± 0.3**	**97.0 ± 0.6**
7.36	SER380	5.6 ± 0.5	21.7 ± 0.4	SER366	**60.8 ± 0.6**	**81.7 ± 1.4**
7.39	THR383	**99.6 ± 0.1**	**100.0 ± 0.1**	THR369	**100.0 ± 0.0**	**100.0 ± 0.0**
7.43	TYR387	**99.7 ± 0.1**	**99.9 ± 0.1**	TYR373	**98.1 ± 0.4**	**97.1 ± 0.2**
