# Supplementary material for: The E2.65A mutation disrupts dynamic binding poses of SB269652 at the dopamine D2 and D3 receptors
Source: PLoS Comput Biol. 2018 Jan 16;14(1):e1005948. doi: 10.1371/journal.pcbi.1005948 (PMC5786319; doi:10.1371/journal.pcbi.1005948)
Supplement: S2 Table — Ballesteros-Weinstein numbering for the subsegments is given in parenthesis. (PDF) [file pcbi.1005948.s012.pdf]

S2 Table. Definition of helical subsegments. Ballesteros-Weinstein numbering for the subsegments is given in parenthesis.

**Subsegments**	**D2R**	**D3R**
**NT**	1 - 30	1 - 26
**TM1e (1.29 - 1.36)**	31 - 38	27 - 33
**TM1m (1.37 - 1.45)**	39 - 47	34 - 42
**TM1i (1.46 - 1.60)**	48 - 62	43 - 57
**IL1**	63 - 67	58 - 62
**TM2e (2.62 - 2.66)**	92 - 96	87 - 91
**TM2m (2.52 - 2.61)**	82 - 91	77 - 86
**TM2i (2.38 - 2.51)**	68 - 81	63 - 76
**EL1**	97 - 103	92 - 99
**TM3e (3.22 - 3.31)**	104 - 113	100 - 109
**TM3m (3.32 - 3.38)**	114 - 120	110 - 116
**TM3i (3.39 - 3.56)**	121 - 138	117 - 134
**IL2**	139 - 146	135 - 144
**TM4e (4.56 - 4.62)**	166 - 172	164 - 170
**TM4m (4.50 - 4.55)**	160 - 165	158 - 163
**TM4i (4.37 - 4.49)**	147 - 159	145 - 157
**EL2**	173 - 186	171 - 185
**TM5e (5.36 - 5.44)**	187 - 195	186 - 194
**TM5m (5.45 - 5.52)**	196 - 203	195 - 202
**TM5i (5.53 - 5.68)**	204 - 219	203 - 218
**IL3**	220 - 336	219 - 321
**TM6e (6.55 - 6.60)**	364 - 369	349 - 354
**TM6m (6.47 - 6.54)**	356 - 363	341 - 348
**TM6i (6.28 - 6.46)**	337 - 355	322 - 340
**EL3**	370 - 375	355 - 361
**TM7e (7.32 - 7.38)**	376 - 382	362 - 368
**TM7m (7.39 - 7.46)**	383 - 390	369 - 376
**TM7i (7.47 - 7.55)**	391 - 399	377 - 385
**H8**	400 - 414	386 - 400
