# Supplementary material for: The E2.65A mutation disrupts dynamic binding poses of SB269652 at the dopamine D2 and D3 receptors
Source: PLoS Comput Biol. 2018 Jan 16;14(1):e1005948. doi: 10.1371/journal.pcbi.1005948 (PMC5786319; doi:10.1371/journal.pcbi.1005948)
Supplement: S3 Table — 3 sets of 500 Bayesian Markov model samples were subjected to RMSD based clustering of the NT conformation. The percentage populations of the three largest clusters are shown, the populations of these clusters in the most dominant MS in each condition are shown in bold. The numbers in parenthesis refer to cluster ids. (PDF) [file pcbi.1005948.s013.pdf]

S3 Table. RMSD based clustering of the N terminus region. 3 sets of 500 Bayesian Markov model samples were subjected to RMSD based clustering of the NT conformation. The percentage populations of the three largest clusters are shown, the populations of these clusters in the most dominant MS in each condition are shown in bold. The numbers in parenthesis refer to cluster ids.

**Largest cluster**	**D2R/WT**	**D2R/E$^{2.65}$A**	**D3R/WT**	**D3R/E$^{2.65}$A**
**1st**	18.1 ± 1.1<br>**17.6 ± 0.8**<br>(3)	43.5 ± 2.4<br>**69.9 ± 4.1**<br>(3)	18.3 ± 6.3<br>**21.5 ± 8.8**<br>(5)	41.4 ± 8.9<br>**35.2 ± 10.8**<br>(9)
**2nd**	18.0 ± 3.5<br>**17.8 ± 5.8**<br>(5)	11.4 ± 0.9<br>**2.6 ± 0.9**<br>(6)	14.8 ± 1.0<br>**7.1 ± 1.1**<br>(18)	11.7 ± 1.1<br>**13.7 ± 0.3**<br>(1)
**3rd**	17.7 ± 0.4<br>**0.8 ± 0.8**<br>(12)	11.1 ± 1.3<br>**7.0 ± 1.9**<br>(2)	8.9 ± 4.7<br>**7.3 ± 5.6**<br>(6)	6.2 ± 5.8<br>**8.0 ± 7.7**<br>(14)
